# Bioactive Polyoxygenated Steroids from the South China Sea Soft Coral, *Sarcophyton* sp

**DOI:** 10.3390/md11030775

**Published:** 2013-03-11

**Authors:** Zenglei Wang, Hua Tang, Pan Wang, Wei Gong, Mei Xue, Hongwei Zhang, Taofang Liu, Baoshu Liu, Yanghua Yi, Wen Zhang

**Affiliations:** Research Center for Marine Drugs, School of Pharmacy, Second Military Medical University, 325 Guohe Road, Shanghai 200433, China; E-Mails: wangzenglei@gmail.com (Z.W.); tanghua0309@126.com (H.T.); w.p1008@hotmail.com (P.W.); gongweicn@foxmail.com (W.G.); bubble1206@hotmail.com (M.X.); hanbing1977.88@hotmail.com (H.Z.); liu5012004008@sina.com (T.L.); liubaoshu@126.com (B.L.)

**Keywords:** *Sarcophyton* sp., steroid, bioactivity, antibacterial, antifungal

## Abstract

Seven new polyoxygenated steroids (**1**–**7**) were isolated together with seven known analogues (**8**–**14**) from the South China Sea soft coral, *Sarcophyton* sp. The structures of the new compounds were identified on the basis of extensive spectroscopic analysis and comparison with reported data. All the steroids are characterized with 3β,5α,6β-hydroxy moiety, displaying carbon skeletons of cholestane, ergostane, gorgostane and 23,24-dimethyl cholestane. In the *in vitro* bioassay, metabolites exhibited different levels of antimicrobial activity against bacterial species *Escherichia coli* and *Bacillus megaterium*, and fungal species *Microbotryum violaceum* and *Septoria tritici*. No inhibition was detected towards microalga *Chlorella fusca*. Preliminary structure-activity analysis suggests that the 11α-acetoxy group may increase both antibacterial and antifungal activities. The terminal-double bond and the cyclopropane moiety at the side chain may also contribute to the bioactivity.

## 1. Introduction

Soft corals are thought to produce various bioactive metabolites that chemically defend themselves from attack [1,2,3]. The soft coral *Sarcophyton* species (order Alcyonacea, family Alcyoniidae) are prolific in the South China Sea and are dominant in many coral reef areas [4]. Chemical research on the animals of this genus has established that it is a rich resource of steroids, diterpenes and tetraterpenes [5,6].

**Figure 1:**
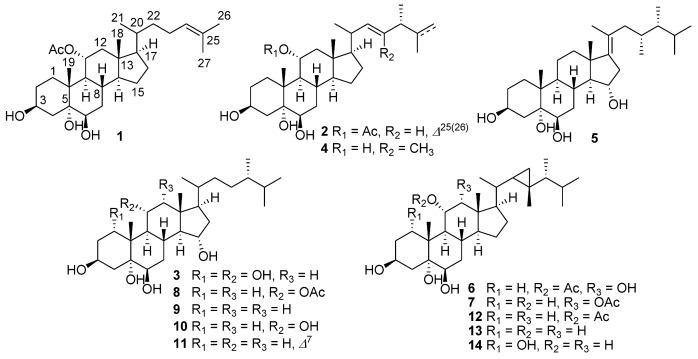
The chemical structures of sterols **1**–**14**.

In the course of our ongoing screening for bioactive secondary metabolites from marine sources [7,8,9,10,11], we have made a collection of *Sarcophyton* sp. off Weizhou Island, Guangxi Province, China. Chemical investigation on the Et_2_O-soluble fraction of the acetone extract from *Sarcophyton* sp. resulted in the isolation of fourteen steroids (**1**–**14**, Figure 1) with 3β,5β,6β-hydroxy moiety. The sterols can be re-sorted into four clusters, due to their different carbon skeletons, namely cholesterol-, ergosterol-, gorgosterol- and 23,24-dimethyl cholesterol-types, displaying an excellent example of chemical diversity. The isolates were tested for *in vitro* antimicrobial activity against bacteria, fungi and a microalga. Here, we describe the isolation, structural elucidation and bioactivity of these new metabolites.

## 2. Results and Discussion

Freshly collected specimens of *Sarcophyton* sp*.* were kept at −20 °C before extraction. The acetone extract of the soft coral was partitioned between Et_2_O and H_2_O. Purification of the Et_2_O extract, by repeated column chromatography on silica and Sephadex LH-20 and followed by reversed-phase semi-preparative HPLC, yielded the pure compounds shown in **1**–**14 **(Figure 1). 

On the basis of detailed spectroscopic analysis and comparison with reported data, the structures of the known compounds were readily determined as (24*S*)-11α-acetoxy-ergostane-3β,5α,6β-triol (**8**, sarcoaldosterol A) [12,13,14], (24*S*)-ergostane-3β,5α,6β-triol (**9**) [15], (24*S*)-ergostane-3β,5α,6β,11α-tetraol (**10**)** [16]**, (24*S*)-ergostane-7-en-3β,5α,6β-triol (**11**) [16], 11α-acetoxy-gorgostane-3β,5α,6β-triol (**12**) [17], gorgostane-3β,5α,6β,11α-tetraol (**13**, sarcoaldosterol B) [13,18] and gorgostane-1α,3β,5α,6β,11α-pentaol (**14**) [19,20,21]. Sarcoaldosterols A (**8**)and B (**13**) were first isolated from the Okinawan soft coral *Sarcophyton* sp. [13]. Later on, sarcoaldosterol A was obtained from the soft corals of genus *Heteroxenia* collected from the Philippines [12] and the Red Sea [14], while sarcoaldosterol B was re-obtained from the Vietnamese sea soft coral, *S. mililatensis* [18]. Compound **9** was reported from the gorgonian *Isis hippuris* [15], a known source of gorgosteroids. Ergosteroids **10** and **11** were first isolated together from the Palauan soft coral, *Lobophytum* cf. *pauciflorum* [16], and then compound **12** was re-isolated from soft coral *S. crassocaule* [21], gorgonian *Plexaurella grisea* [20] and *Junceella juncea* [19]. The gorgosteroid **12** was recently reported from Egyptian Red Sea soft coral *Heteroxenia ghardaqensis* [17], while **14** was repeatedly reported from the scallop *Patinopecten yessoensis* [22] and the sponge *Spongionella gracilis* [23] and *Dysidea fragilis* [24].

Compound **1** was isolated as an optically active, white amorphous solid. The molecular formula was established as C_29_H_48_O_5_ by high-resolution electrospray ionization mass spectrometry (HR-ESI-MS) from the pseudo-molecular ion at *m/z* 499.3397 [M + Na]^+^, indicating six degrees of unsaturation. The infrared (IR) absorption at 3408 cm^−1^ and 1712 cm^−1^ showed the presence of hydroxyl and carbonyl groups in the molecule. This observation was in agreement with the signals in the ^13^C nuclear magnetic resonance (NMR) and distortionless enhancement by polarization transfer (DEPT0 spectra (Table 1) for 3 sp^2^ carbon atoms (1 × OC=O, CH=C) at a lower field and 26 sp^3^ carbon atoms at a higher field (1 × OC, 3 × OCH, 2 × C, 5 × CH, 9 × CH_2_, 6 × CH_3_), accounting for two degrees of unsaturation. The remaining degrees of unsaturation were attributed to the presence of four rings in the molecule.

The NMR spectra of compound **1** were closely related to those of co-occurring (24*S*)-11α-acetoxy-ergostane-3β,5α,6β-triol (**8**), displaying identical signals for rings A, B, C and D. A difference was observed in the nature of the side chain. The methyls of the isopropyl group (*δ*_H_ 0.84, 3H, d, *J* = 6.8 Hz, H_3_-27; 0.76, 3H, d, *J* = 6.8 Hz, H_3_-27) in **8 **were replaced by two vinyl methyls (*δ* 1.59 and 1.67, each 3H, s) in **1** (Table 2). In correspondence, the doublet methyl signal of H_3_-28 (*δ* 0.76, 3H, d, *J* = 6.7 Hz) in **8** was replaced by a trisubstituted double bond linked to a methylene (*δ* 5.07, 1H, br t, *J* = 7.2 Hz) in **1**, indicating a ∆^24^ cholestane side chain instead of an ergostane side chain in the molecule. These data led **1 **to the structure of 11α-acetoxy-cholesta-24-en-3β,5α,6β-triol. The ^1^H NMR and ^13^C NMR assignments of the side chain were identical to those of reported data [25,26,27] and were further confirmed by the proton sequence from H_3_-21 to H-24, as established by the ^1^H-^1^H correlation spectroscopy (COSY) experiment and the heteronuclear multiple bond (HMBC) correlation from H_2_-26 and H_3_-27 to both C-25 and C-24.

Compound **2** was obtained as an optically active, amorphous powder. Its formula was established as C_30_H_48_O_5_ by HR-ESI-MS. The NMR data of **2** also resembled those of **8**, with a recognized difference in the signals assigned to the side chains. The methyls of the isopropyl group (*δ*_H_ 0.84, 3H, d, *J* = 6.8 Hz, H_3_-27; 0.76, 3H, d, *J* = 6.8 Hz, H_3_-27) in **8 **were replaced by a vinyl methyl (*δ* 1.67, 3H, s) and a terminal double bond (*δ* 4.70, 2H, br s) in **2** (Table 2), leading to the assignment of the double bond as ∆^25^. In addition, a pair of signals for a disubstituted double bond were observed (*δ* 5.26, 1H, dd, *J* = 15.4, 6.2 Hz; *δ* 5.20, 1H, dd, *J* = 15.4, 7.3 Hz). The large coupling constant between the olefinic protons indicated an *E* geometry of the double bond, which was consequently assigned as ∆^22^. The marked-down field shift of H_3_-21 and H_3_-28 from *δ* 0.87 and 0.76 in **8** to *δ* 0.98 and 1.07 in **2** supported the above conclusion. Further structural evidence came from the proton sequence from H_3_-21 to H_3_-27, as established by the ^1^H-^1^H COSY experiment and the HMBC correlation from H_3_-28 to C-23, C-24 and C-25, H_2_-26 and H_3_-27 to both C-25 and C-24. The ^1^H NMR assignments of the side chain were identical to those of (22*E*,24*S*)-ergostane-5,22,25-trien-3β-ol, isolated from the plant, *Clerodendrum fragrans* (Verbenaceae family) [28], which further confirmed the established structure and suggested an *S* configuration at C-24. These lines of evidence led to the structure (22*E*,24*S*)-11α-acetoxy-ergostane-22,25-dien-3β,5α,6β-triol for compound **2**.

**Table 1:** The ^13^C nuclear magnetic resonance (NMR) data of sterols **1**–**7 **(100 *^a^* or 125 *^b^* MHz in CDCl_3_).

Carbon	1 *^a^*	2 *^a^*	3 *^b^*	4 *^a^*	5 *^a^*	6 *^a^*	7 *^a^*
1	33.5 t	33.5 t	76.8 d	34.4 t	32.3 t	33.7 t	33.9 t
2	31.3 t	31.3 t	37.2 t	31.2 t	30.9 t	31.3 t	31.2 t
3	67.2 d	67.2 d	64.2 d	67.4 d	67.6 d	67.1 d	67.5 d
4	41.3 t	41.3 t	41.3 t	41.0 t	40.8 t	41.3 t	41.3 t
5	76.6 s	76.6 s	76.8 s	76.7 s	76.7 s	76.6 s	76.7 s
6	76.1 d	76.1 d	75.2 d	76.1 d	76.1 d	76.0 d	76.0 d
7	34.8 t	34.9 t	35.2 t	34.5 t	34.8 t	34.4 t	34.0 t
8	29.4 d	29.4 d	29.4 d	29.1 d	28.9 d	29.4 d	28.9 d
9	48.6 d	48.7 d	46.6 d	53.0 d	45.9 d	42.7 d	46.3 d
10	39.9 s	39.9 s	42.1 s	40.0 s	38.4 s	39.6 s	39.6 s
11	71.1 d	71.1 d	68.0 d	68.7 d	21.5 t	73.4 d	70.7 d
12	46.4 t	46.3 t	50.9 t	51.8 t	37.9 t	75.0 d	80.5 d
13	42.9 s	42.7 s	43.2 s	43.1 s	44.7 s	46.7 s	46.0 s
14	54.6 d	54.7 d	54.7 d	55.1 d	52.2 d	45.3 d	47.4 d
15	24.2 t	24.2 t	24.2 t	24.2 t	72.2 d	23.9 t	23.8 t
16	28.2 t	28.5 t	28.2 t	28.0 t	34.4 t	27.5 t	27.6 t
17	56.0 d	55.9 d	55.8 d	56.7 d	149.0 s	49.2 d	50.1 d
18	12.7 q	12.9 q	12.8 q	13.4 q	17.3 q	11.8 q	12.7 q
19	16.8 q	16.8 q	16.3 q	17.0 q	16.8 q	16.7 q	16.7 q
20	35.4 d	39.8 d	36.1 d	34.5 d	133.0 s	35.1 d	34.6 d
21	18.6 q	20.7 q	18.9 q	20.6 q	17.1 q	20.1 q	20.6 q
22	35.9 t	135.5 d	33.6 t	131.3 d	42.0 t	31.9 d	31.9 d
23	24.7 t	131.8 d	30.6 t	135.7 s	32.7 d	25.9 s	25.9 s
24	125.1 d	43.6 d	39.1 d	50.2 d	44.2 d	50.8 d	50.8 d
25	131.1 s	149.8 s	31.5 d	30.8 d	30.1 d	32.0 d	32.0 d
26	17.6 q	108.8 t	20.5 q	21.7 q	21.6 q	21.5 q	21.5 q
27	25.7 q	20.6 q	17.6 q	20.1 q	19.1 q	22.2 q	21.3 q
28		18.9 q	15.5 q	17.0 q	11.5 q	15.4 q	15.5 q
29				13.3 q	13.8 q	14.3 q	14.3 q
30						21.3 t	21.3 t
COOCH_3_	22.1 q	22.1 q				22.1 q	22.2 q
COOCH_3_	170.4 s	170.4 s				170.0 s	170.4 s

s = singlet; d = doublet, t = triplet; q = quartet.

**Table 2:** The ^1^H NMR data of sterols **1**–**4** (400 *^a^* or 500 *^b^* MHz in CDCl_3_; *J* in Hz).

Position	1 *^a^*	2 *^a^*	3 *^b^*	4 *^a^*
1	1.26 (m), 1.81( m)	1.22 (m), 1.76 (m)	4.11 (br s)	1.50 (m), 1.71 (m)
2	1.47 (m), 1.82 (m)	1.53 (m), 1.77 (m)	1.86 (m), 2.18 (m)	1.56 (m), 1.86 (m)
3	4.05 (m)	4.05 (m)	4.40 (m)	4.07 (m)
4	1.62 (ov), 2.07 (t, 12.2)	1.58 (ov), 2.08 (d, 11.8)	1.77 (ov), 2.08 (ov)	1.67 (ov), 2.11 (ov)
6	3.52 (br s)	3.52 (br s)	3.51 (br s)	3.53 (br s)
7	1.61 (m), 1.76 (m)	1.58 (m), 1.74 (m)	1.52 (m), 1.88 (m)	1.73 (m), 2.08 (m)
8	1.87 (m)	1.76 (m)	1.88 (m)	1.76 (m)
9	1.74 (m)	1.82 (m)	1.99 (m)	1.34 (m)
11	5.14 (dd, 10.3, 5.4)	5.14 (dd, 10.5, 5.4)	4.01 (11.0, 5.5)	3.94 (9.7, 4.9)
12	1.16 (ov)	1.18 (ov)	1.26 (ov)	1.19 (ov)
	2.31 (dd, 12.2, 5.4)	2.30 (dd, 12.1, 5.4)	2.33 (dd, 11.5, 5.5)	2.34 (11.5, 4.9)
14	1.28 (m)	1.18 (m)	1.19 (m)	1.16 (m)
15	1.06 (m), 1.56 (m)	1.07 (m), 1.79 (m)	1.09 (m), 1.60 (m)	1.03 (m), 1.59 (m)
16	1.20 (m), 1.88 (m)	1.26 (m), 1.50 (m)	1.26 (m), 1.90 (m)	1.19 (m), 1.78 (m)
17	1.15 (m)	1.18 (m)	1.19 (m)	1.19 (m)
18	0.73 (s)	0.75 (s)	0.66 (s)	0.73 (s)
19	1.28 (s)	1.28 (s)	1.21 (s)	1.32 (s)
20	1.38 (m)	2.00 (m)	1.37 (m)	2.30 (m)
21	0.90 (d, 6.5)	0.98 (d, 6.6)	0.94 (d, 6.5)	0.92 (d, 6.9)
22	1.02 (m), 1.39 (m)	5.26 (dd, 15.4, 6.2)	0.89 (m), 1.39 (m)	4.87 (d, 9.5)
23	1.87 (m), 1.99 (m)	5.20 (dd, 15.4, 7.3)	0.89 (m), 1.38 (m)	
24	5.07 (t, 7.2)	2.69 (m)	1.19 (m)	1.67 (m)
25			1.60 (m)	1.53 (m)
26	1.59 (s)	4.70 (br s)	0.86 (d, 7.0)	0.84 (d, 6.5)
27	1.67 (s)	1.67 (s)	0.79 (d, 7.0)	0.78 (d, 6.5)
28		1.07 (d, 6.9)	0.78 (d, 7.0)	0.93 (d, 7.1)
29				1.50 (s)
COO CH_3_	1.98 (s)	1.98 (s)		

s = singlet; d = doublet; t = triplet; m = multiplet; br s = broad singlet; dd = doublet of doublets; ov = overlapped signals.

Compound **3** was also obtained as an optically active, amorphous powder. The HR-ESI-MS of compound **3** exhibited a pseudo-molecular ion peak at *m/z* 489.4582 [M + Na]^+^, leading to the determination of the molecular formula as C_28_H_50_O_5_. Analysis of the NMR spectra of **3** (Table 1 and Table 2) revealed a remarkable similarity to those of the co-occurring sterol **10**, except for the replacement of a methylene group (*δ*_H_ 1.49, 1H, m, H-1α 1.73, 1H, m, H-1β; *δ*_C_ 34.4, t) in **10** by a secondary hydroxyl group (*δ*_H_ 4.11, 1H, br s; *δ*_C_ 76.8, d) in **3**. This hydroxyl group was assigned at C-1, due to the proton connectivity from H-1 to H_2_-4, as established by the ^1^H-^1^H COSY spectrum and the obvious HMBC correlations from H_3_-19 to C-1, C-5, C-9 and C-10. A β configuration of H-1 was deduced from its coupling constant pattern (br s) and supported by the observation of NOE interactions between H_3_-19 and H-1. A comparison of the NMR data for the core structure of **3** with those of co-isolated gorgostane-1α,3β,5α,6β,11α-pentaol (**14**) [22,23,24] further confirmed the established structure. The absolute configuration at C-24 was determined as *S*, due to the ^1^H NMR shift values of H_3_-21 (*δ* 0.936, s), H_3_-27 (*δ* 0.790, s) and H_3_-26 (*δ* 0.856, s), similar to those reported for **8**–**11** [13,16,18,19,20,21], instead of those for the C-24 epimer (*δ* 0.925, s, H_3_-21; 0.805, s, H_3_-27; 0.853, s, H_3_-26) [23]. The structure of **3** was evidently established as (24*S*)-ergostane-1α,3β,5α,6β,11α-pentaol.

Furthermore, compound **4** was also obtained as an optically active, amorphous powder. Its formula C_29_H_50_O_4_ was deduced from the molecular ion at *m/z* 485.3532 [M + Na]^+^ in the HR-ESI-MS spectrum. A comparison of the ^1^H and ^13^C NMR spectra of **4** (Table 1 and Table 2) and the co-isolated **10** revealed a great similarity with the exception of an appearance of a trisubstituted double bond (*δ*_H_ 4.87, d, *J* = 9.5 Hz; *δ*_C_ 131.3, d, 135.7, s) and an additional vinylic methyl group (*δ*_H_ 1.50, s) in the side chain. The double bond was placed at C-22, due to the proton spin system of H_3_-21/H-20/H-22 and the long-range correlations of H_3_-29/C-22, C-23 and C-24, as deduced from the ^1^H-^1^H COSY and the HMBC spectra, respectively. The downfield shift of H_3_-28 from *δ* 0.77 in **10** to *δ* 0.93 in **4** supported the above conclusion. The ^1^H NMR and ^13^C NMR assignments of the side chain were identical to those of model compounds [29,30,31,32,33,34]. An *S* absolute stereochemistry at C-24 in **4** was proposed by careful comparison of ^1^H NMR data with those of in dinosterol and its C-24 epimer [31], where the ^1^H signals for H_3_-21 (*δ* 0.921, d, *J* = 6.9 Hz)), H_3_-27 (*δ* 0.779, d, *J* = 6.8 Hz) and H_3_-26(*δ* 0.836, d, *J* = 6.5 Hz) were close to those of dinosterol (*δ* 0.919, d, *J* = 6.5 Hz, H_3_-21; 0.778, d, *J* = 6.5 Hz, H_3_-26; 0.837, d, *J* = 6.5 Hz, H_3_-27) rather than its C-24 epimer (*δ* 0.910, d, *J* = 6.8 Hz, H_3_-21; 0.772, d, *J* = 6.8 Hz, H_3_-26; 0.848, d, *J* = 6.5 Hz, H_3_-27). Therefore, the structure of **4** is (24*S*)-23,24-dimethylcholesta-22-en-3β,5α,6β,11α-tetraol.

Compound **5**, an optically active, amorphous powder, had the same molecular formula (C_29_H_50_O_4_) as that of **4**, as deduced by HR-ESI-MS. Its NMR spectra also showed the characteristic signals for the 3β,5α,6β-trihydroxyl moiety. However, the trisubstituted double bond (*δ*_H_ 4.87, d, *J* = 9.5 Hz; *δ*_C_ 131.3, d, 135.7, s) in **4** was replaced by a tetrasubstituted double bond (*δ* 149.0, s; 133.0, s) in **5** (Table 1 and Table 3). The double bond was readily assigned as ∆^17(20)^, due to the observation of the vinyl methyl singulate for H_3_-21 (*δ* 1.71, s) and its HMBC correlation with C-17, C-20 and C-22, and the HMBC correlation from H_3_-18 to C-17 provided further evidence. Meanwhile, the secondary alcohol at C-11 had to be assigned to C-15, due to the proton sequence from H-9 to H_2_-12 and from H-14 to H_2_-16, as established by the ^1^H-^1^H COSY experiment. The location of 15-OH was confirmed by the HMBC correlation from H-15 to both C-13 and C-17. The diagnostic interactions between H_3_-18 and H-15 in the NOESY spectrum indicated a β configuration of the proton. The *R* configuration at both C-23 and C-24 were suggested to be the same, biogenetically, as those in sarcosterol, 25-hydroxy sarcosterol and peridinosterol, produced by the soft corals, *S. glaucum* [35] and *Sinularia mayi* [36] and the symbiotic dinoflagellate [37], respectively. Biosynthetic studies suggested the same C-23 stereochemistry in sarcosterol and peridinosterol, due to the symbiotic relationship of soft coral and dinoflagellate [36]. The structure of compound **5** was tentatively determined to be (23*R*,24*R*)-23,24-dimethylcholesta-17(20)-en-3β,5α,6β-triol. 

Compounds **6**, **7** and **12**–**14 **belong to the cluster of gorgosteroids, which are characterized as having a cyclopropane ring in the side chain. Compound **6** was isolated as an optically active, white amorphous solid. The molecular formula was established as C_32_H_54_O_6_ by HR-ESI-MS. The NMR data of **6** were almost identical to those of **12**, displaying characteristic proton signals for the cyclopropane ring at the very high field (*δ*_H_ −0.13~0.46) (Table 3) in the ^1^H NMR spectrum. One methylene group (*δ*_H_ 2.33, dd, *J* = 12.2, 5.3 Hz, H-12β and 1.17, m, H-12α; *δ*_C_ 46.5) in **12** was replaced by a oxygenated methine group (*δ*_C_ 75.0, CH; *δ*_H_ 3.95, d, *J* = 3.0 Hz, 1H) in **6**. This hydroxyl group was assigned at C-12, due to HMBC correlation from H_3_-18 to H-12 and the proton connectivity of H-9/H-11/H-12. The β configuration of H-12 was deduced from its small coupling constant with H-11 and its nuclear Overhauser effect (NOE) effect with H_3_-18. The structure of **6** was thus elucidated as 11α-acetoxy-gorgostane-3β,5α,6β,12α-tetraol.

**Table 3:** The ^1^H NMR data of sterols **5**–**8** (400 *^a^* or 500 *^b^* Hz in CDCl_3_; *J* values are in Hz).

Position	5 *^a^*	6 *^a^*	7 *^a^*
1	1.43 (m), 1.58 (m)	1.27 (m), 1.80 (m)	1.63 (m), 2.16 (m)
2	1.55 (m), 1.82 (m)	1.52 (m), 1.81 (m)	1.47 (m), 1.85 (m)
3	4.11 (m)	4.06 (m)	4.05 (m)
4	1.63 (ov), 2.09 (ov)	1.61 (ov), 2.10 (ov)	1.63 (ov), 2.15 (ov)
6	3.59 (br s)	3.52 (br s)	3.54 (br s)
7	1.50 (m), 1.69 (m)	1.53 (m), 1.76 (m)	1.60 (m), 1.74 (m)
8	1.72 (m)	1.84 (m)	1.85 (m)
9	1.40 (m)	2.08 (m)	1.62 (m)
11	1.40 (m), 1.49 (m)	5.27 (dd, 11.4, 3.0)	4.12 (dd, 10.2, 3.0)
12	1.51 (m), 2.35 (m)	3.95 (d, 3.0)	5.27 (d, 3.0)
14	1.74 (m)	1.88 (m)	1.72 (m)
15	4.64 (d, 5.0)	1.08 (m), 1.61 (m)	1.12 (m), 1.63 (m)
16	1.65 (m), 1.78 (m)	1.31 (m), 2.05 (m)	1.39 (m), 2.00 (m)
17		1.96 (m)	1.68 (m)
18	0.90 (s)	0.76 (s)	0.79 (s)
19	1.20 (s)	1.27 (s)	1.29 (s)
20		1.02 (m)	1.01 (m)
21	1.71 (s)	1.01 (br s)	0.93 (d, 6.5)
22	1.95(dd, 13.3, 4.3),	0.23 (ov)	0.13 (ddd, 9.3, 6.1, 5.7)
	2.25 (dd, 12.7, 9.5)		
23	1.80 (m)		
24	1.05 (m)	0.23 (ov)	0.24 (m)
25	1.62 (m)	1.50 (m)	1.63 (m)
26	0.83 (d, 6.7)	0.85 (d, 6.5)	0.85 (d, 6.9)
27	0.89 (d, 8.1)	0.94 (d, 6.5)	0.95 (d, 6.5)
28	0.79 (d, 6.9)	0.93 (d, 6.7)	0.94 (d, 6.4)
29	0.70 (d, 6.8)	0.89 (s)	0.89 (s)
30		0.46 (dd, 8.8, 4.2),	0.46 (dd, 8.7, 4.4),
		−0.14 (d, 5.0)	−0.13 (d, 5.0)
COO CH_3_		2.08 (s)	2.17 (s)

s = singlet; d = doublet; t = triplet; m = multiplet; br s = broad singlet; dd = doublet of doublets; ov = overlapped signals.

Compound **7** was isolated as an optically active, white amorphous solid. The compound had the same formula (C_32_H_54_O_6_) as **6** on the basis of HR-ESI-MS. Inspection of NMR spectra for **7** revealed great similarity with those of **6** concerning the signals in rings A, C, D and the side chain. Analysis of the ^1^H-^1^H COSY spectrum for the proton sequence H-9/H-11/H-12 and the HMBC correlation from H_3_-18 to C-12 led the assignment of 12-OAc and 11-OH in **7** instead of 11-OAc and 12-OH in **6**. The configuration at chiral centers remains intact due to the analysis of related proton coupling constants and the NOE effects, establishing the structure of **7** as 12α-acetoxy-gorgostane-3β,5α,6β,11α-tetraol. 

The isolation of an array of sterols demonstrates an excellent example of chemical diversity, displaying carbon skeletons of cholestane, ergostane, gorgostane and 23,24-dimethyl cholestane. The co-occurrence of these metabolites supported the biosynthesis proposal of side chains in dinosterol, peridinosterol and gorgosterol [32]. The methylation of the side chain of **1** may yield the side chain of **2**, which further would be methylated to the side chain of the dinosterol derivative **4** and the peridinosterol analogues, **5** and **6**. The side chain of **4** was then methylated to the side chain of sterols belonging to the gorgosterol family (**6**, **7**, **12**–**14**).

All isolated compounds, except **5**, were tested in an agar diffusion assay for their antibacterial, antifungal and algicidal properties (Table 4). All the metabolites exhibited antibacterial activity against the Gram-negative bacterium, *Escherichia coli*, the Gram-positive bacterium, *Bacillus megaterium*, and antifungal activity against the fungi, *Microbotryum violaceum* and *Septoria tritici*. However, **1**, **2**, **6**, **7 **and **8** exhibited considerable growth inhibitory activity with regard to bacteria and/or fungi species. This suggests that the 11*α*-acetoxy group may increase both antibacterial and antifungal activities. The terminal-double bond and the cyclopropane side chain seem to also contribute to this bioactivity. None of the sterols showed inhibition of green alga *Chlorella fusca*.

**Table 4:** Agar diffusion assays for antibacterial, antifungal and antialgal activities ^a,b^.

Compound	*Escherichia coli*	*Bacillus megaterium*	*Microbotryum violaceum*	*Septoria tritici*	*Botrytis cinerea*	*Chlorella fusca*
**1**	12.0	10.0	6.5	10.5	10.5	0
**2**	14.5	12.0	10.0	7.5	5.5	0
**3**	7.5	4.5	7.0	10.5	5.5	0
**4**	6.0	7.0	7.5	9.0	0	0
**6**	9.5	10.0	8.5	10.0	8.0	0
**7**	8.0	10.0	7.0	4.5	12.0	0
**8**	7.0	7.5	6.0	6.5	0	0
**9**	4.5	4.5	7.0	6.5	0	0
**1** **0**	6.0	8.5	8.5	4.5	0	0
**1** **1**	6.0	7.0	6.0	6.0	0	0
**1** **2**	7.0	8.0	9.5	10.0	5.0	0
**1** **3**	10.0	6.5	11.5	7.5	0	0
**1** **4**	5.0	4.0	8.5	7.0	0	0
Penicillin	17.5	22.5	11.5	9.0	0	0
Strepolin	8.0	7.5	6.5	9.0	0	0
Ketoconazole	10.0	10.0	20.5	20.0	26.5	0
Acetone	0	0	0	0	0	0

^a ^0.05 mg of the test or control substances dissolved in acetone were applied to a filter disc and sprayed with the respective test organism at a concentration of 10^5^ cells/mL. ^b ^Radii of the zones of inhibition are given in mm (gi = growth inhibition); that is, some growth within the zone of inhibition. Otherwise, the inhibition zone was clear.

## 3. Experimental Section

### 3.1. General Experimental Procedures

Commercial silica gel (200–300 mesh, 10–40 mm; Yantai, China) and Sephadex LH-20 (Pharmacia) were used for column chromatography. Precoated silica gel plates (Yantai, GF_254_ plate, 10–40 mm) were used for analytical thin-layer chromatography (TLC). Spots were visualized by heating Si gel plates sprayed with 10% H_2_SO_4_ in EtOH. Semipreparative HPLC was carried out on an Agilent 1100 liquid chromatography equipped with a refractive index detector using a Zorbax 300 SB-C18 column (25 cm × 9.4 mm i.d.). Melting points were determined on an XT5-XMT apparatus. Optical rotations were measured with a Perkin-Elmer 341 polarimeter. NMR spectra were recorded in CHCl_3_ on a Bruker DRX 400 or 500 spectrometer, and the 2D NMR spectra were obtained using standard pulse sequences. Chemical shifts are reported in parts per million (*δ*), with use of the residual CHCl_3_ signal (*δ*_H_ 7.26 ppm) as an internal standard for ^1^H NMR and CDCl_3_ (*δ*_C_ 77.0 ppm) for ^13^C NMR; Coupling constants (*J*) are reported in Hz. ^1^H NMR and ^13^C NMR assignments were complemented by heteronuclear single quantum correlation (HSQC), HMBC, ^1^H-^1^H COSY and nuclear Overhauser effect spectroscopy (NOESY) experiments. The following abbreviations were used to describe spin multiplicity: s = singlet; d = doublet; t = triplet; q = quartet; m = multiplet; br s = broad singlet; dd = doublet of doublets; ov = overlapped signals. HR-ESI-MS were recorded on a Micromass Quattro mass spectrometer. Optical rotations were measured in CHCl_3_ with an Autopol IV polarimeter at the sodium D line (590 nm). Infrared spectra were recorded in thin polymer films on a Nexus 470 FTIR spectrophotometer (Nicolet, USA); peaks are reported in cm^−1^.

### 3.2. Material and Methods

The soft coral *Sarcophyton* sp. was collected by hand using SCUBA off the coast of Weizhou Island, Guangxi Province of China, in October 2008, at a depth of 20 m and identified by Dr. Xiu-Bao Li, South China Sea Institute of Oceanology, Chinese Academy of Sciences. A voucher specimen (No. S-7-1) was deposited at the Research Center for Marine Drugs, School of Pharmacy, Second Military Medical University, Shanghai, China.

### 3.3. Extraction and Isolation

The frozen bodies of *Sarcophyton* sp. (1642 g, wet weight) were cut into small pieces, exhaustively extracted with acetone (5 L × 5) and then evaporated under reduced pressure. The acetone extract was resuspended in H_2_O and partitioned with Et_2_O 5 times. The concentrated Et_2_O extract (21.6 g) was subjected to column chromatography (CC) on a Si gel using petroleum ether/acetone (99:1–1:10) as eluent to yield seven fractions (Fr. A–G) on the basis of thin layer chromatography (TLC) analysis. Fractions D–F were gel-filtered on Sephadex LH-20 (*n*-hexane/CH_2_Cl_2_/MeOH, 2:1:1), followed by CC on Si gel eluting with gradient CHCl_3_/MeOH (50:1, 30:1, 20:1, 10:1) and further purification by semipreparative HPLC to yield **1** (2.3 mg, 28.4 min, 95% MeOH, 1.0 mL/min), **2** (3.3 mg, 29.6 min, 95% MeOH, 1.0 mL/min), **8** (33.7 mg, 33.9 min, 95% MeOH, 1.0 mL/min), **9** (25.5 mg, 31.2 min, 96% MeOH, 1.5 mL/min), **11** (1.2 mg, 25.1 min, 96% MeOH, 1.5 mL/min) and **12** (5.4 mg, 38.8 min, 95% MeOH, 1.0 mL/min) from fraction D, **3** (3.4 mg, 16.2 min, 95% MeOH, 1.0 mL/min), **6** (3.4 mg, 15.3 min, 95% MeOH, 1.0 mL/min) and **14** (0.5mg, 16.7 min, 95% MeOH, 1.0 mL/min) from fraction E and **4** (1.4 mg, 17.4 min), **5** (13.2 mg, 20.1 min), **7** (9.5 mg, 18.7 min), **10** (11.4 mg, 18.0 min) and **13** (4.8 mg, 20.8 min) from fraction F (94% MeOH, 1.5 mL/min).

Compound **1**: white amorphous solid; melting point (m.p.) 108–110 °C; [*α*]_D_^20^ −34.5 (*c* 0.165, CHCl_3_); IR (film) ν_max_ 3408 (board), 2927, 2869, 1712, 1376, 1263, 1027, 757; ^1^H and ^13^C-NMR data, see Table 1 and Table 2; HRESI-MS *m/z*: 499.3397 [M + Na]^+^ (calculated (calcd.) C_29_H_48_O_5_Na, 499.3399).

Compound **2**: white amorphous solid; m.p. 173–175 °C; [*α*]_D_^20^ −44.3 (*c* 0.115, CHCl_3_); IR (film) ν_max_ 3401 (board), 2961, 2927, 2869, 1715, 1375, 1216, 1026, 801; ^1^H and ^13^C-NMR data, see Table 1 and Table 2; HRESI-MS *m/z*: 511.3403 [M + Na]^+^ (calcd*.* C_30_H_48_O_5_Na, 511.3399). 

Compound **3**: white amorphous solid; m.p. 264–265 °C; [α]_D_^20^ −35.3 (*c* 0.085, CHCl_3_); IR (film) ν_max_ 3362 (board), 2956, 2927, 2870, 1464, 1378, 1067, 1042; ^1^H and ^13^C-NMR data, see Table 1 and Table 2; HRESI-MS *m/z*: 467.3731 [M + H]^+^ (calcd. C_28_H_50_O_5_Na, 467.3737).

Compound **4**: white amorphous solid; m.p. 245–247 °C; [α]_D_^20^ −38.9 (*c* 0.095, CHCl_3_); IR (film) ν_max_ 3412 (board), 2960, 2926, 2869, 1462, 1261, 1096, 801; ^1^H and ^13^C-NMR data, see Table 1 and Table 2; HRESI-MS *m/z*: 485.3606 [M + Na]^+^ (calcd. C_29_H_50_O_4_Na, 485.3707).

Compound **5**: white amorphous solid; m.p. 150–152 °C; [α]_D_^20^ −12.1 (*c* 0.165, CHCl_3_); ^1^H and ^13^C-NMR data, see Table 1 and Table 3; HRESI-MS *m/z*: 485.3609 [M + Na]^+^ (calcd. C_29_H_50_O_4_Na, 485.3607).

Compound **6**: white amorphous solid; m.p. 170–172 °C; [α]_D_^20^ −20.0 (*c* 0.17, CHCl_3_); IR (film) ν_max_ 3434 (board), 2957, 2931, 2874, 1716, 1460, 1372, 1255; ^1^H and ^13^C-NMR data, see Table 1 and Table 3; HRESI-MS *m/z*: 557.3819 [M + Na]^+^ (calcd. C_32_H_54_O_6_Na, 557.3818).

Compound **7**: white amorphous solid; m.p. 150–152 °C; [α]_D_^20^ −9.8 (*c* 0.395, CHCl_3_); IR (film) ν_max_ 3422, 2956, 2926, 2872, 1719, 1459, 1375, 1254; ^1^H and ^13^C-NMR data, see Table 1 and Table 3; HRESI-MS *m/z*: 557.3829 [M + Na]^+^ (calcd. C_32_H_54_O_6_Na, 557.3818).

### 3.4. Agar Diffusion Test for Biological Activity

An agar diffusion test was applied to determine the biological activities, as previously reported [38]. Briefly, compounds were dissolved in acetone at 1.0 mg/mL (2.1 mM for **1**, 2.0 mM for **2**, 2.3 mM for **3**, 2.2 mM for **4** and **5**, 1.9 mM for **6** and **7**, 2.0 mM for **8**, 2.3 mM for **9**, 2.2 mM for **10**, 2.3 mM for **11**, 1.9 mM for **12**, 2.1 mM for **13** and 2.0 mM for **14**); 50 μL of the solution (0.05 mg) were pipetted onto a sterile filter disc (Schleicher & Schuell, 9 mm), which was placed onto an appropriate agar growth medium for the respective test organism and subsequently sprayed with a suspension of the test organism at a concentration of 10^5^ cells/mL. The test organisms were bacteria, the Gram-negative bacterium, *Escherichia coli*, and the Gram-positive bacterium, *Bacillus megaterium* (both grown on NB medium), and fungi, *Microbotryum violaceum*, *Septoria tritici* and *Botrytis cinerea* (all grown on MPY medium), and the microalga, *Chlorella fusca* (grown on CP medium). Penicillin (1.0 mg/mL, 0.05 mg), strepolin (1.0 mg/mL, 0.05 mg) and ketoconazole (1.0 mg/mL, 0.05 mg) were used as positive controls. These microorganisms were chosen because (a) they are non-pathogenic and (b) had in the past proved to be accurate as initial test organisms for antibacterial and antifungal activities [38,39]. Commencing at the outer edge of the filter disc, the radius of the zone of inhibition was measured in mm.

## 4. Conclusions

Chemical investigation of the soft coral *Sarcophyton* sp. from the South China Sea led to the isolation and structural elucidation of fourteen polyoxygenated steroids (**1**–**14**) with 3β,5α,6β-hydroxy moiety, demonstrating an excellent example of chemical diversity. These compounds displayed different levels of antibacterial and antifungal bioactivities in the *in vitro* bioassay. Preliminary structure-activity analysis suggests that the 11α-acetoxy group may increase both antibacterial and antifungal activities. The terminal-double bond and the cyclopropane moiety at the side chain may also contribute to the bioactivity. The interesting discovery may encourage further investigations on the sterols, the antibacterial and antifungal activity, and the structure-activity relationship. 

## Supplementary Files

Supplementary File 1 Supplementary Materials (PDF, 2362 KB)
