# Supplementary material for: Bioactive Polyoxygenated Steroids from the South China Sea Soft Coral, Sarcophyton sp
Source: Mar Drugs. 2013 Mar 11;11(3):775–87. doi: 10.3390/md11030775 (PMC3705369; doi:10.3390/md11030775)

Supplementary Materials

Index

**1**	Index	1–2
**2**	Spectra of the new compound **1**.	3–9
	**Figure S1.** HR-ESIMS spectrum of the new compound **1**	3
	**Figure S2.** ^{1}H NMR spectrum of the new compound **1**	3
	**Figure S3.** ^{13}C NMR spectrum of the new compound **1**	4
	**Figure S4.** DEPT spectrum of the new compound **1**	4
	**Figure S5.** HSQC spectrum of the new compound **1**	5
	**Figure S6.** ^{1}H-^{1}H COSY spectrum of the new compound **1**	6
	**Figure S7.** HMBC spectrum of the new compound **1**	7
	**Figure S8.** NOESY spectrum of the new compound **1**	8
**3**	Spectra of the new compound **2**	9–14
	**Figure S9.** HR-ESIMS spectrum of the new compound **2**	9
	**Figure S10.** ^{1}H NMR spectrum of the new compound **2**	9
	**Figure S11.** ^{13}C NMR spectrum of the new compound **2**	10
	**Figure S12.** DEPT spectrum of the new compound **2**	10
	**Figure S13.** HSQC spectrum of the new compound **2**	11
	**Figure S14.** ^{1}H-^{1}H COSY spectrum of the new compound **2**	12
	**Figure S15.** HMBC spectrum of the new compound **2**	13
	**Figure S16.** NOESY spectrum of the new compound **2**	14
**4**	Spectra of the new compound **3**	15–20
	**Figure S17.** HR-ESIMS spectrum of the new compound **3**	15
	**Figure S18.** ^{1}H NMR spectrum of the new compound **3**	15
	**Figure S19.** ^{13}C NMR spectrum of the new compound **3**	16
	**Figure S20.** DEPT spectrum of the new compound **3**	16
	**Figure S21.** HSQC spectrum of the new compound **3**	17
	**Figure S22.** ^{1}H-^{1}H COSY spectrum of the new compound **3**	18
	**Figure S23.** HMBC spectrum of the new compound **3**	19
	**Figure S24.** NOESY spectrum of the new compound **3**	20
**5**	Spectra of the new compound **4**	21–26
	**Figure S25.** HR-ESIMS spectrum of the new compound **4**	21
	**Figure S26.** ^{1}H NMR spectrum of the new compound **4**	21
	**Figure S27.** ^{13}C NMR spectrum of the new compound **4**	22
	**Figure S28.** DEPT spectrum of the new compound **4**	22
	**Figure S29.** HSQC spectrum of the new compound **4**	23
	**Figure S30.** ^{1}H-^{1}H COSY spectrum of the new compound **4**	24
	**Figure S31.** HMBC spectrum of the new compound **4**	25
	**Figure S32.** NOESY spectrum of the new compound **4**	26
**6**	Spectra of the new compound **5**	27–32
	**Figure S33.** HR-ESIMS spectrum of the new compound **5**	27
	**Figure S34.** ^{1}H NMR spectrum of the new compound **5**	27

Figure S35. ^{13}C NMR spectrum of the new compound **5** 28
Figure S36. DEPT spectrum (1) of the new compound **5** 28
Figure S37. DEPT spectrum (2) of the new compound **5** 29
Figure S38. ^{1}H-^{1}H COSY spectrum of the new compound **5** 29
Figure S39. HSQC spectrum of the new compound **5** 30
Figure S40. HMBC spectrum of the new compound **5** 31
Figure S41. NOESY spectrum of the new compound **5** 32
7 Spectra of the new compound **6** 33–38
Figure S42. HR-ESIMS spectrum of the new compound **6** 33
Figure S43. ^{1}H NMR spectrum of the new compound **6** 33
Figure S44. ^{13}C NMR spectrum of the new compound **6** 34
Figure S45. DEPT spectrum of the new compound **6** 34
Figure S46. HSQC spectrum of the new compound **6** 35
Figure S47. ^{1}H-^{1}H COSY spectrum of the new compound **6** 36
Figure S48. HMBC spectrum of the new compound **6** 37
Figure S49. NOESY spectrum of the new compound **6** 38
8 Spectra of the new compound **7** 39–44
Figure S50. HR-ESIMS spectrum of the new compound **7** 39
Figure S51. ^{1}H NMR spectrum of the new compound **7** 39
Figure S52. ^{13}C NMR spectrum of the new compound **7** 40
Figure S53. DEPT spectrum of the new compound **7** 40
Figure S54. HSQC spectrum of the new compound **7** 41
Figure S55. ^{1}H-^{1}H COSY spectrum of the new compound **7** 42
Figure S56. HMBC spectrum of the new compound **7** 43
Figure S57. NOESY spectrum of the new compound **7** 44

Figure S1. HR-ESIMS spectrum of the new compound **1**.

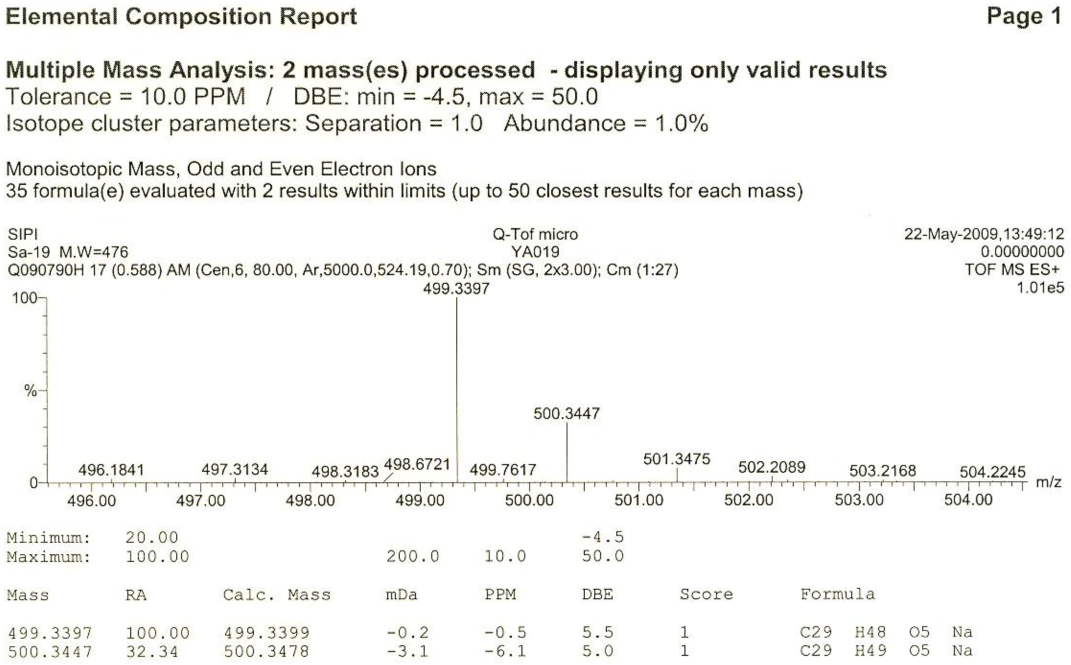

Elemental Composition Report Page 1

Multiple Mass Analysis: 2 mass(es) processed - displaying only valid results
Tolerance = 10.0 PPM / DBE: min = -4.5, max = 50.0
Isotope cluster parameters: Separation = 1.0 Abundance = 1.0%

Monoisotopic Mass, Odd and Even Electron Ions
35 formula(e) evaluated with 2 results within limits (up to 50 closest results for each mass)

SIPI Q-Tof micro 22-May-2009,13:49:12
Sa-19 M.W=476 YA019 0.00000000
Q090790H 17 (0.588) AM (Cen,6, 80.00, Ar,5000.0,524.19,0.70); Sm (SG, 2x3.00); Cm (1:27) TOF MS ES+ 1.01e5

Minimum:	20.00				-4.5		
Maximum:	100.00		200.0	10.0	50.0		
Mass	RA	Calc. Mass	mDa	PPM	DBE	Score	Formula
499.3397	100.00	499.3399	-0.2	-0.5	5.5	1	C29 H48 O5 Na
500.3447	32.34	500.3478	-3.1	-6.1	5.0	1	C29 H49 O5 Na

Figure S2. ^{1}H MNR (400 MHz, $CDCl_3$) spectrum of the new compound **1**.

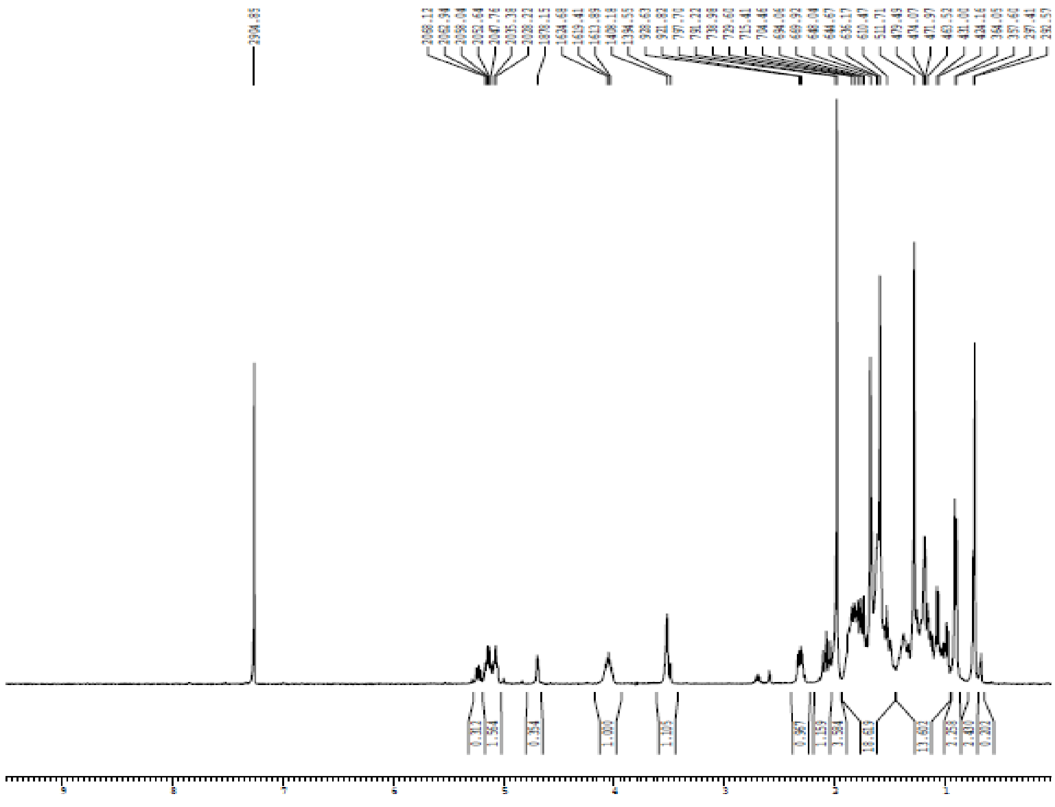

Figure S3. ^{13}C MNR (100 MHz, $CDCl_3$) spectrum of the new compound **1**.

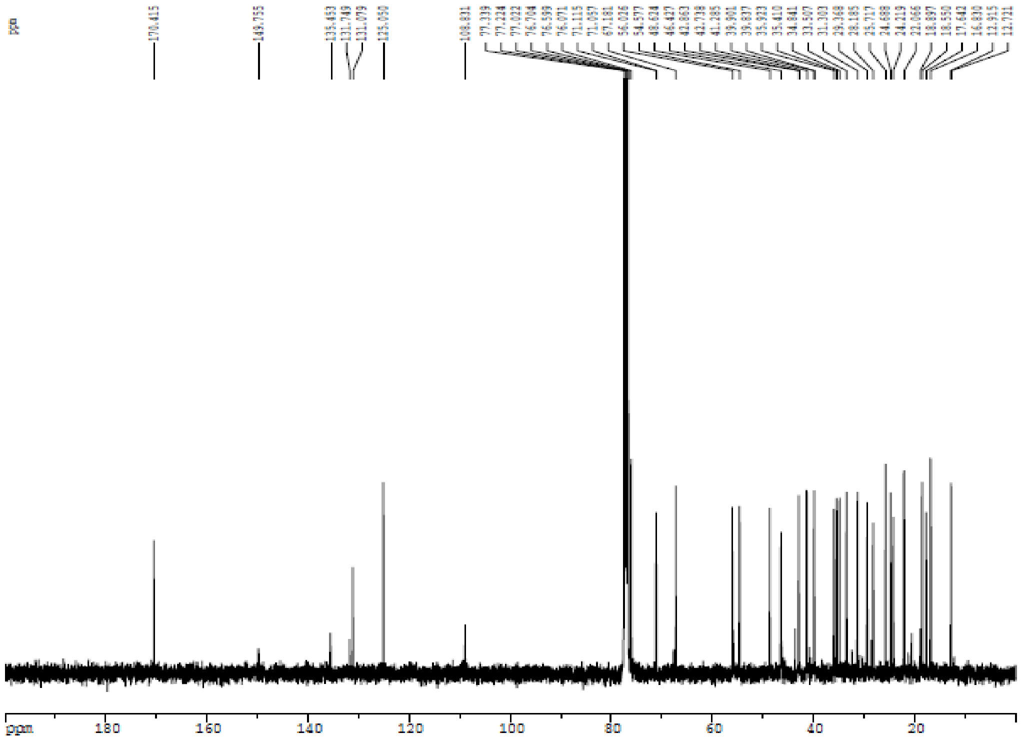

Figure S4. DEPT (100 MHz, $CDCl_3$) spectrum of the new compound **1**.

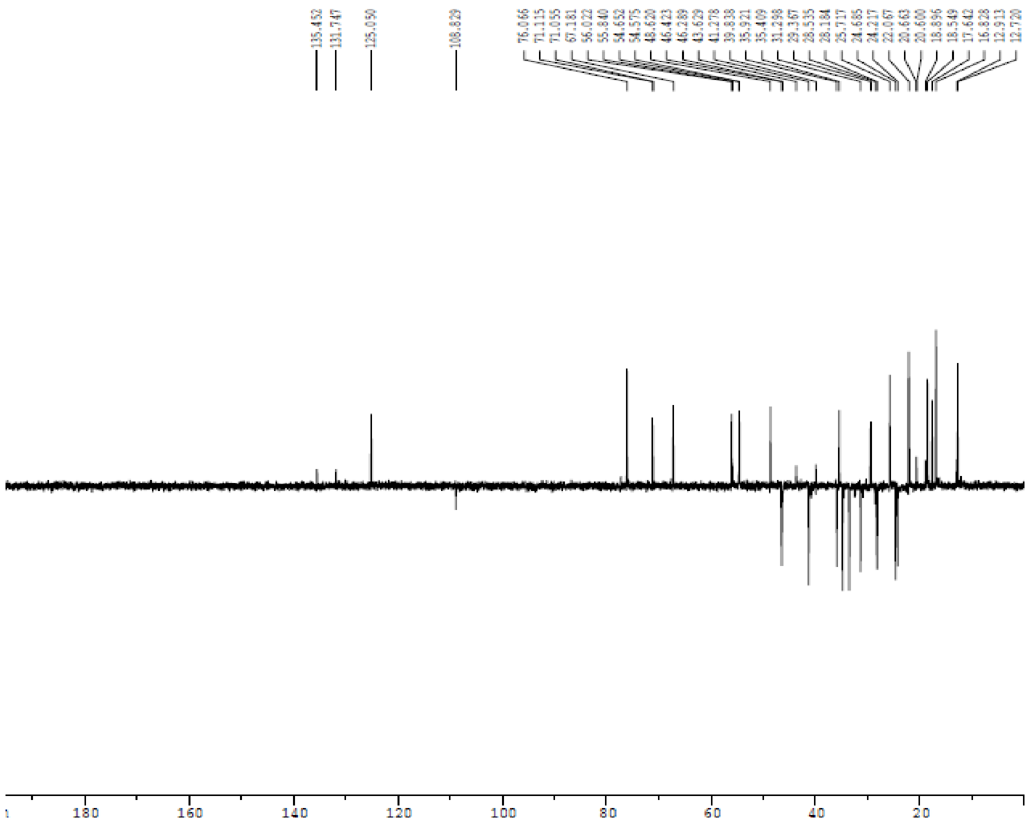

Figure S5. HSQC spectrum of the new compound **1**.

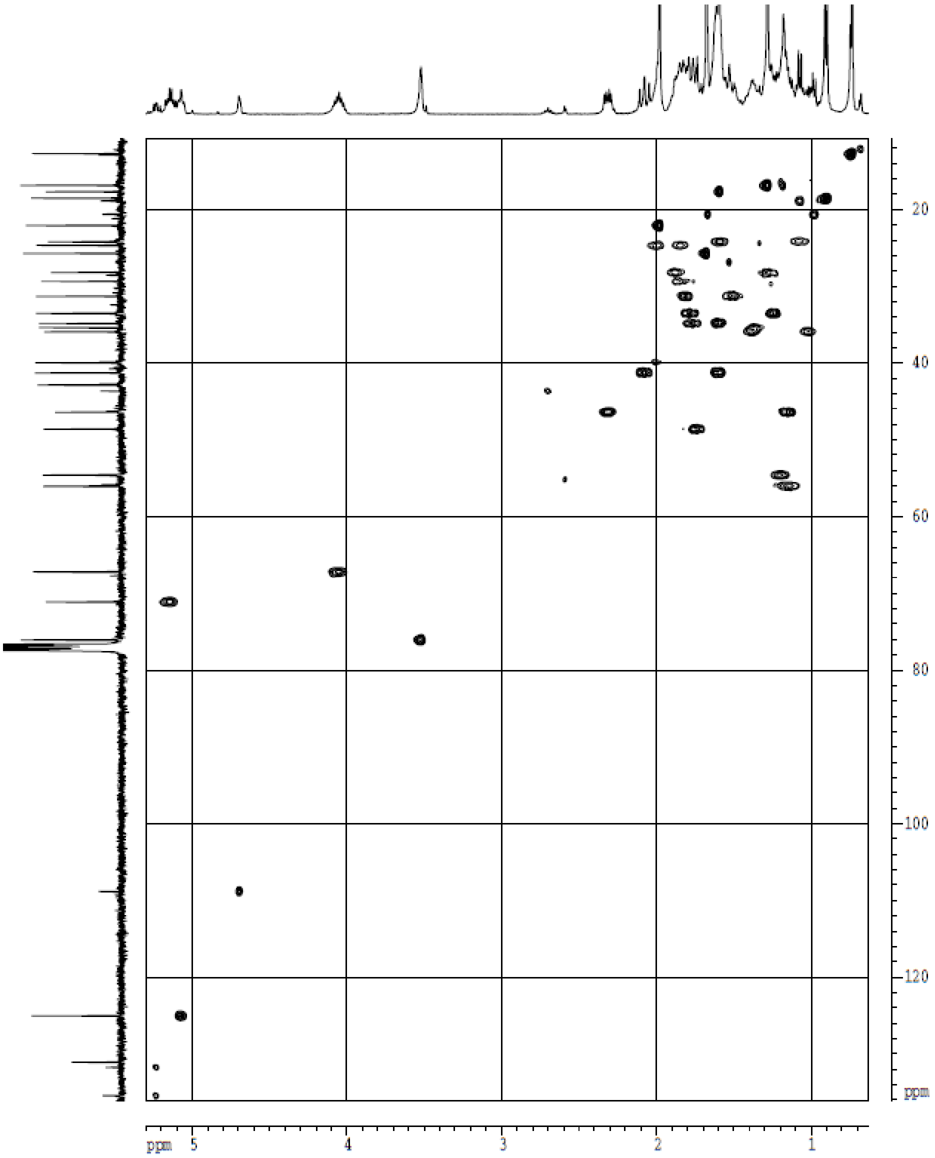

Figure S6. ^{1}H-^{1}H COSY spectrum of the new compound **1**

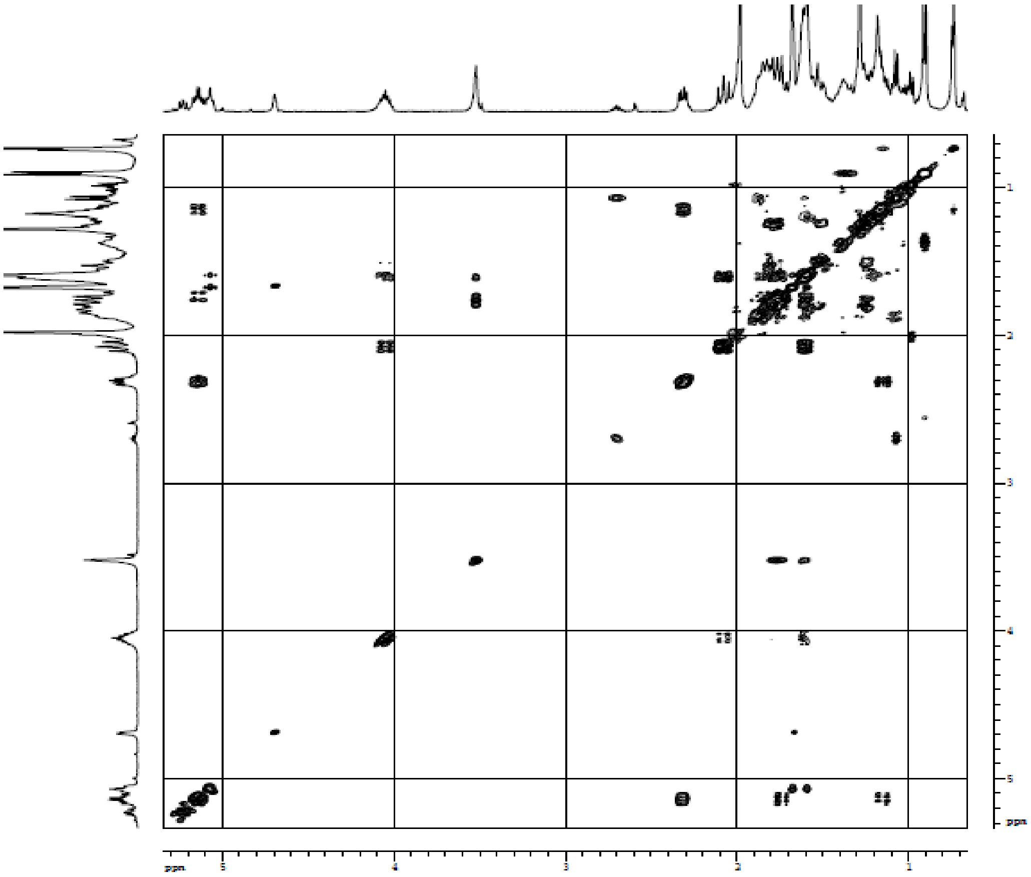

Figure S7. HMBC spectrum of the new compound **1**.

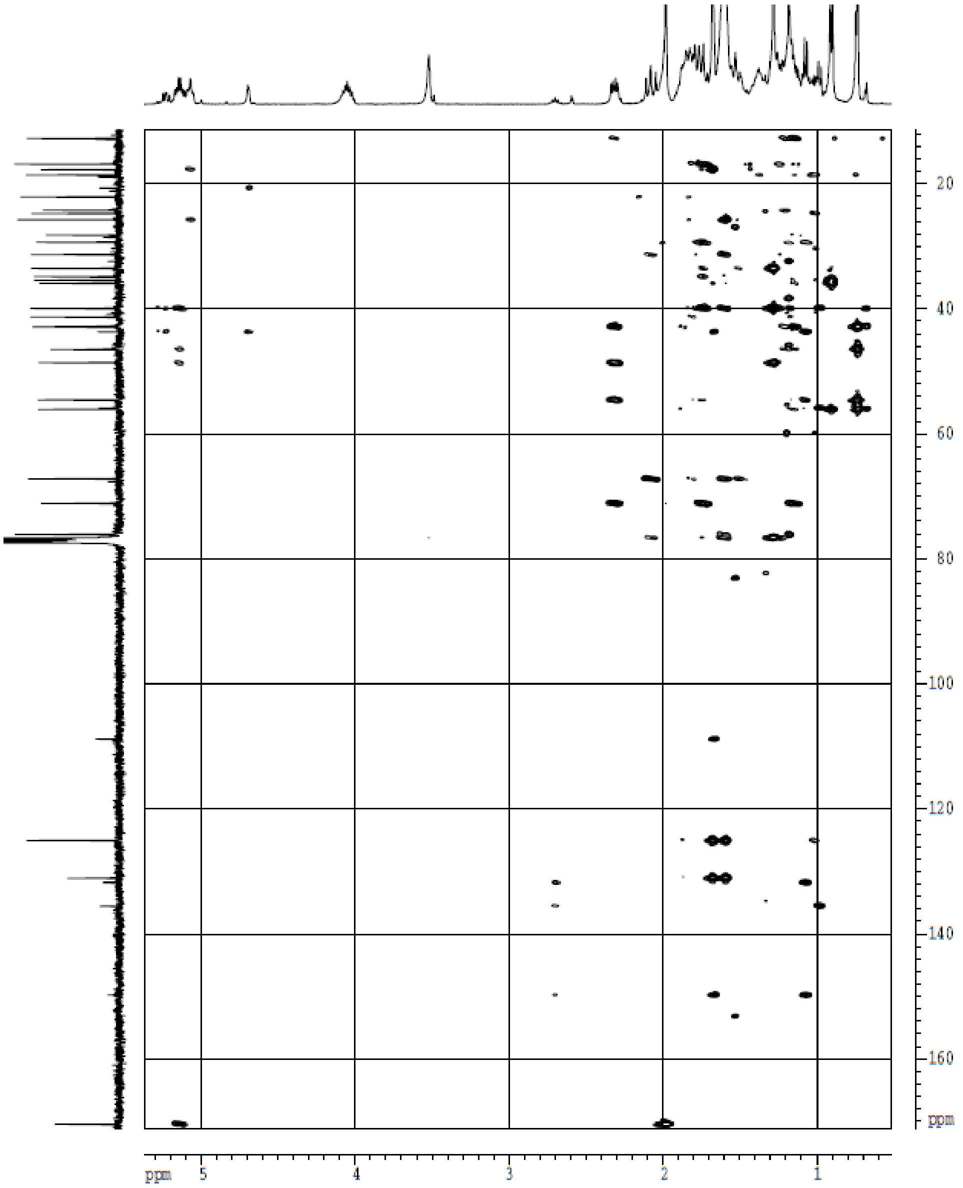

Figure S8. NOESY spectrum of the new compound **1**.

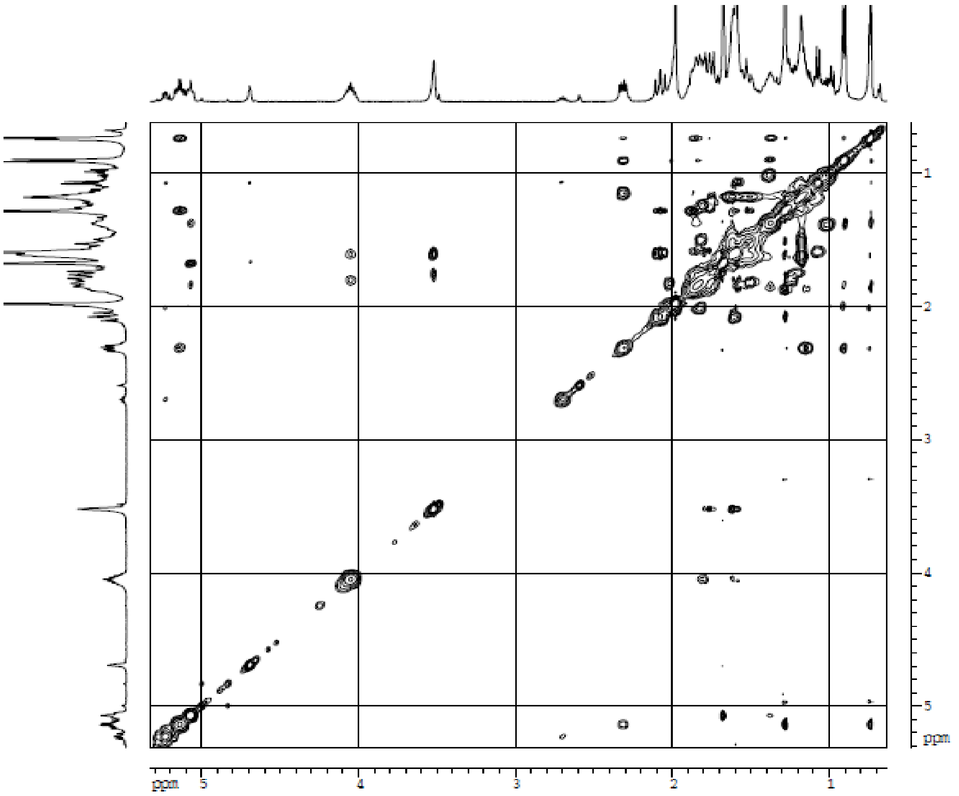

Figure S9. HR-ESIMS spectrum of the new compound **2**.

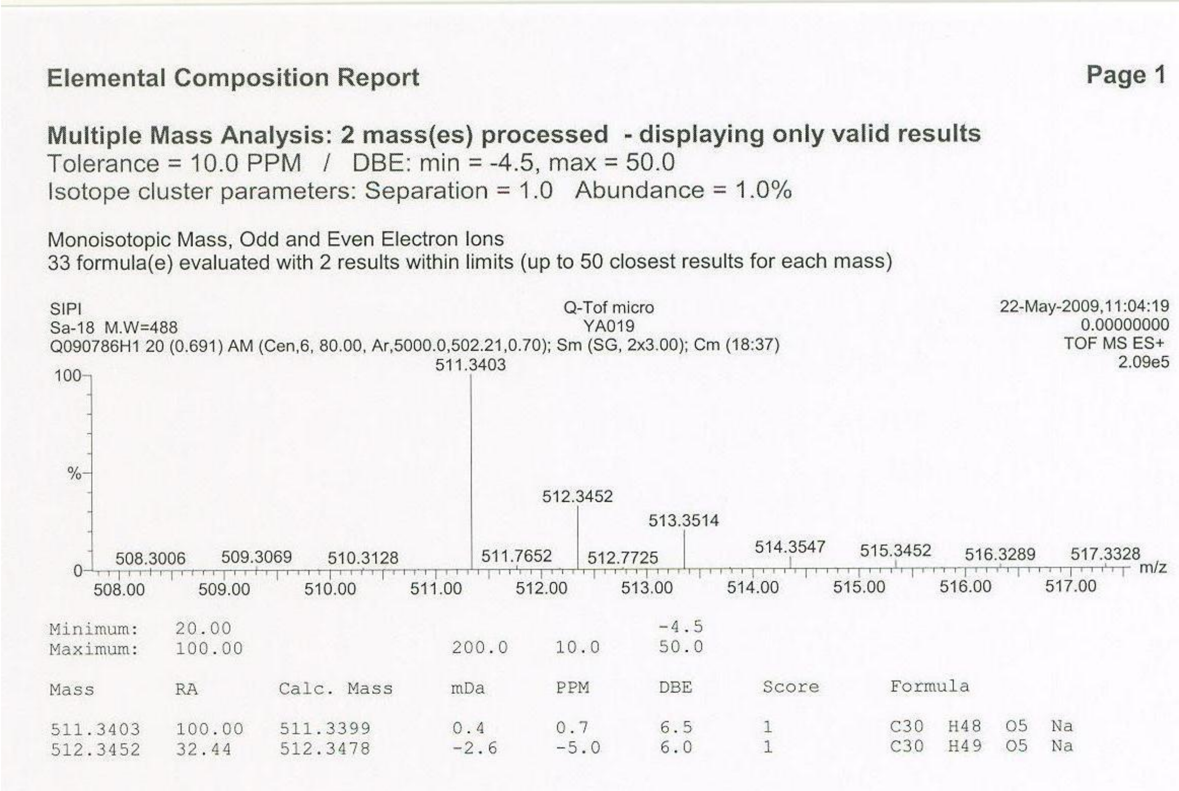

Elemental Composition Report Page 1

Multiple Mass Analysis: 2 mass(es) processed - displaying only valid results
Tolerance = 10.0 PPM / DBE: min = -4.5, max = 50.0
Isotope cluster parameters: Separation = 1.0 Abundance = 1.0%

Monoisotopic Mass, Odd and Even Electron Ions
33 formula(e) evaluated with 2 results within limits (up to 50 closest results for each mass)

SIPI Q-Tof micro 22-May-2009,11:04:19
Sa-18 M.W=488 YA019 0.00000000
Q090786H1 20 (0.691) AM (Cen,6, 80.00, Ar,5000.0,502.21,0.70); Sm (SG, 2x3.00); Cm (18:37) TOF MS ES+ 2.09e5

Mass	RA	Calc. Mass	mDa	PPM	DBE	Score	Formula
Minimum:	20.00				-4.5		
Maximum:	100.00		200.0	10.0	50.0		
511.3403	100.00	511.3399	0.4	0.7	6.5	1	C30 H48 O5 Na
512.3452	32.44	512.3478	-2.6	-5.0	6.0	1	C30 H49 O5 Na

Figure S10. ^{1}H NMR (400 MHz, $CDCl_3$) spectrum of the new compound **2**.

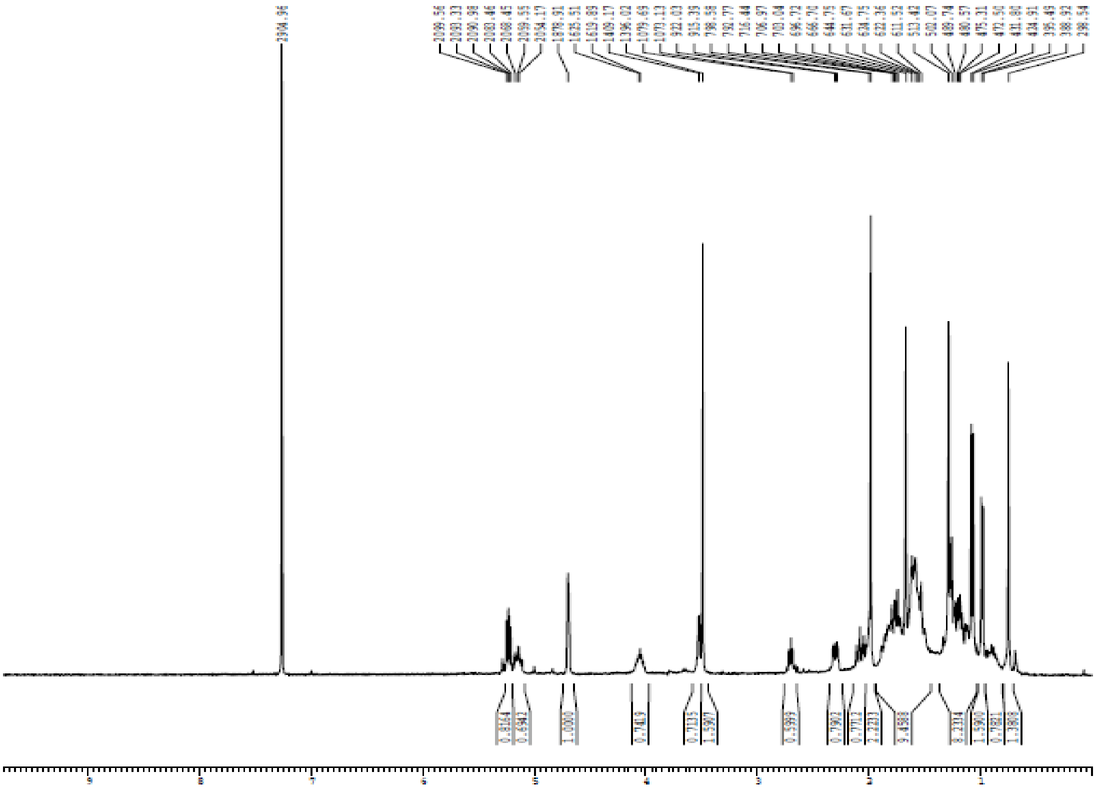

Figure S11. ^{13}C MNR (100 MHz, $CDCl_3$) spectrum of the new compound **2**

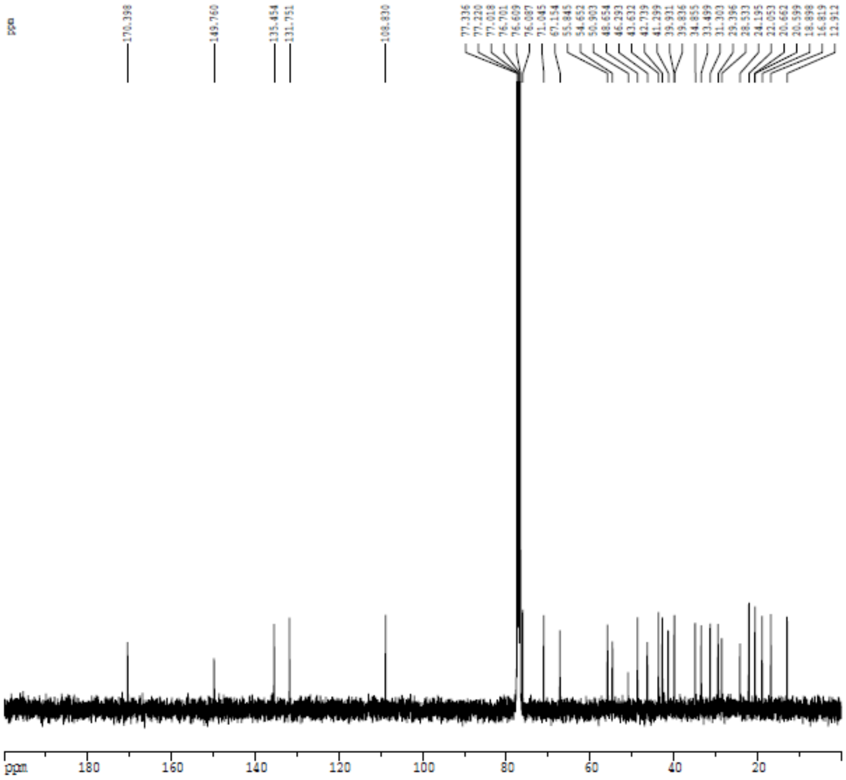

Figure S12. DEPT (100 MHz, $CDCl_3$) spectrum of the new compound **2**.

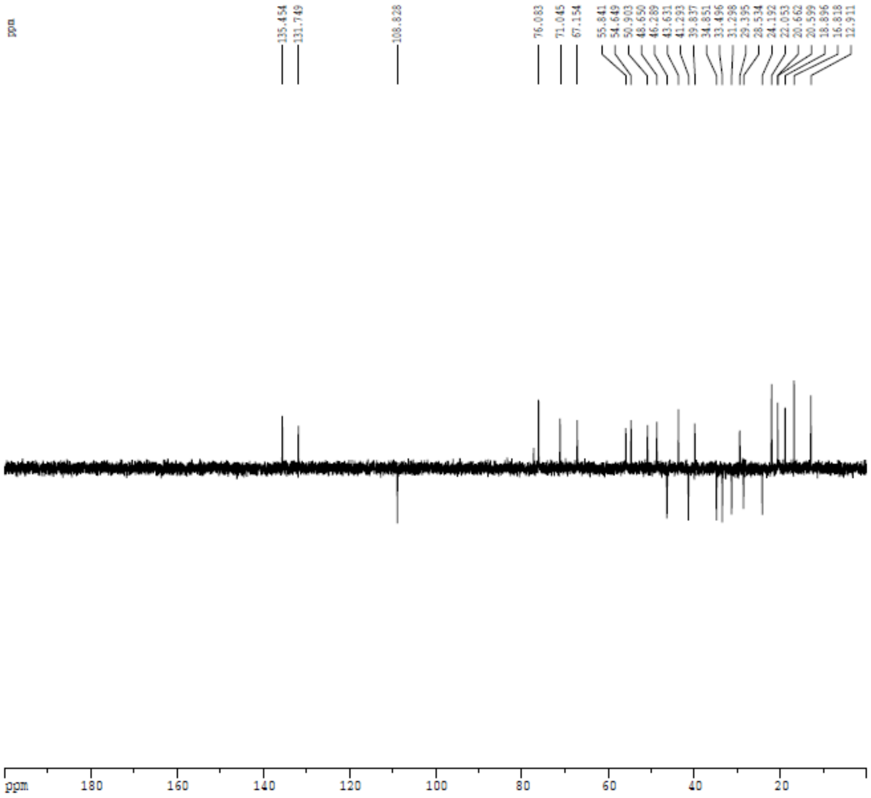

Figure S13. HSQC spectrum of the new compound **2**.

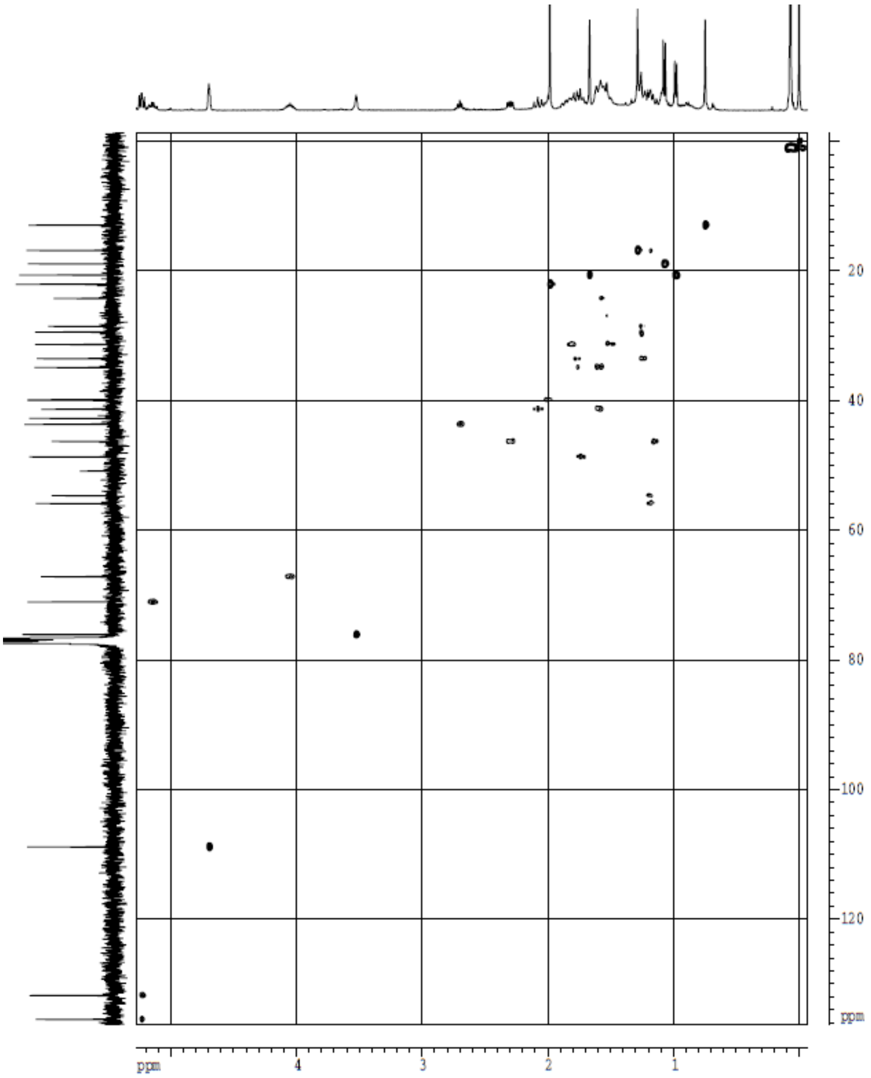

Figure S14. ^{1}H-^{1}H COSY spectrum of the new compound **2**.

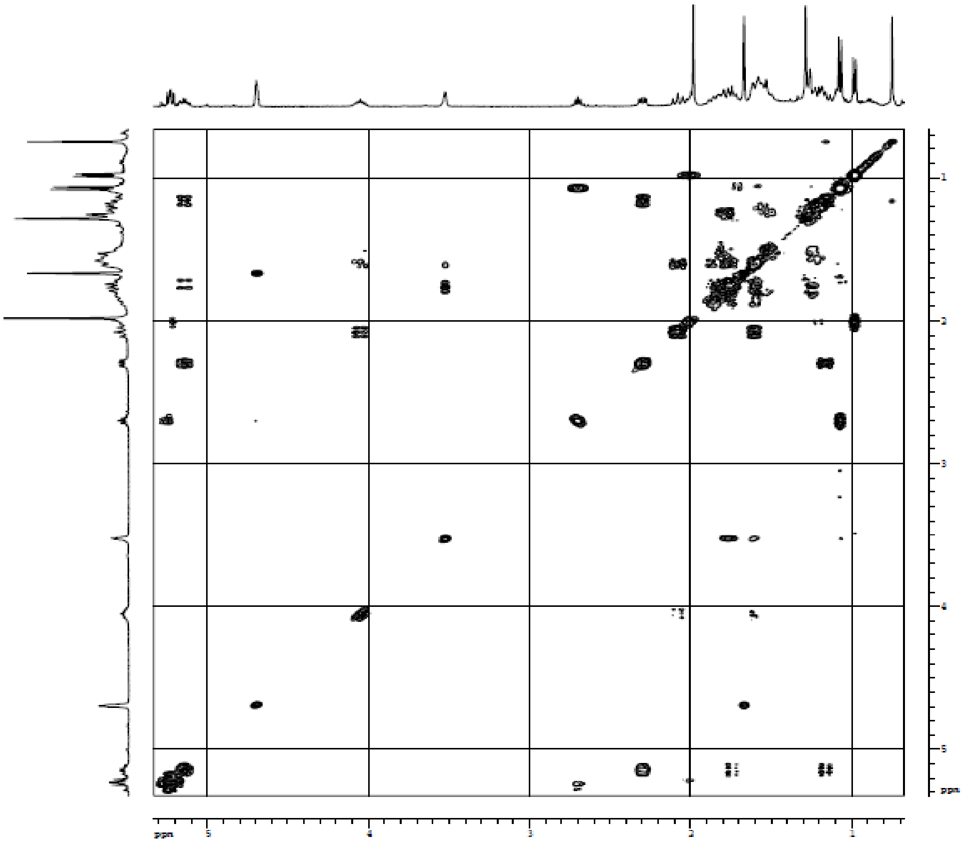

Figure S15. HMBC spectrum of the new compound **2**.

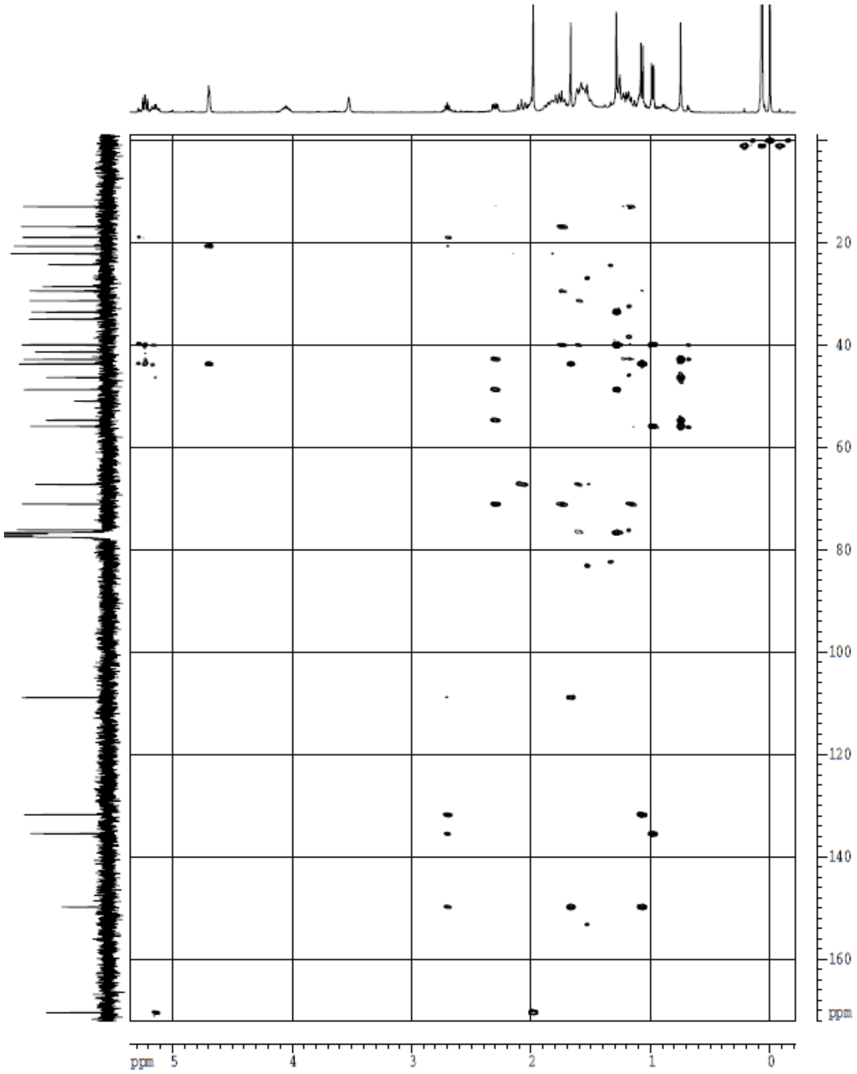

Figure S16. NOESY spectrum of the new compound **2**.

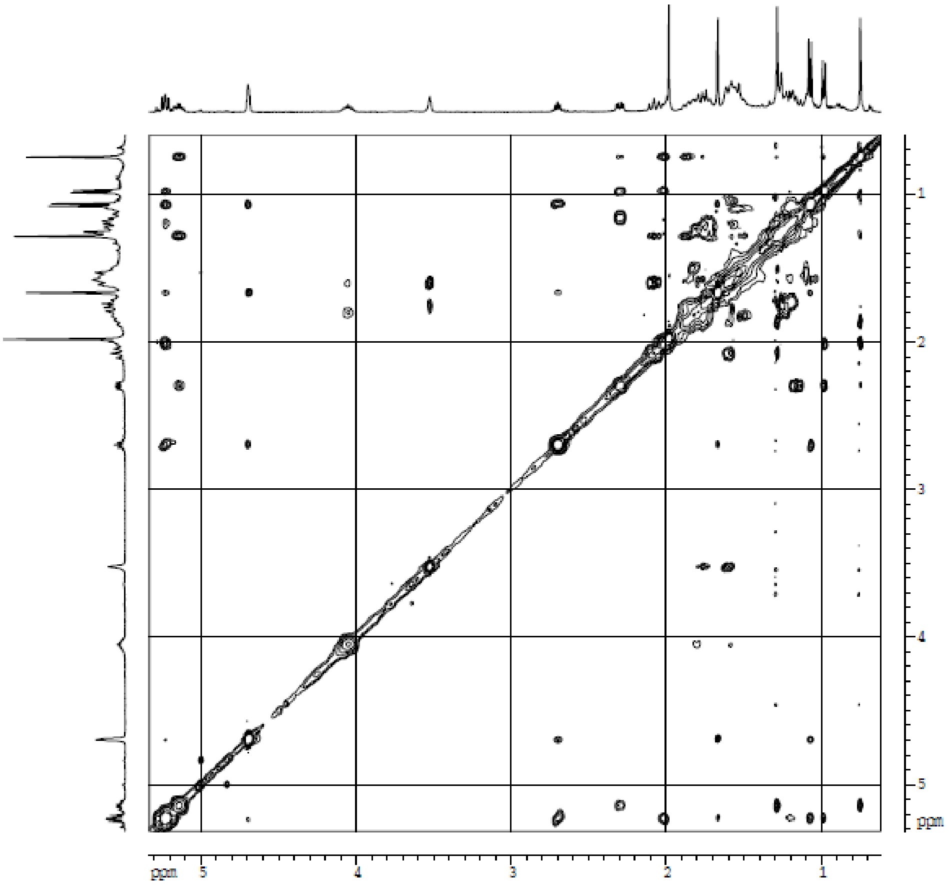

Figure S17. HR-ESIMS spectrum of the new compound **3**.

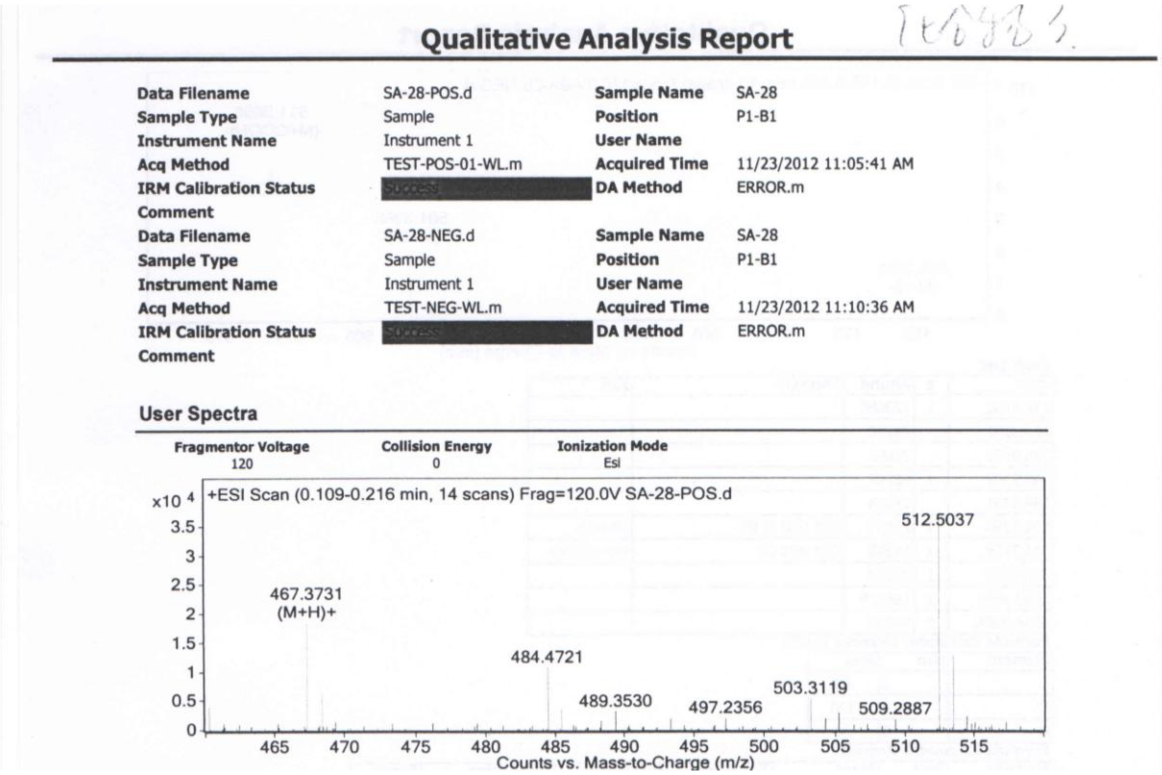

Figure S18. ^{1}H MNR (500 MHz, $CDCl_3$) spectrum of the new compound **3**.

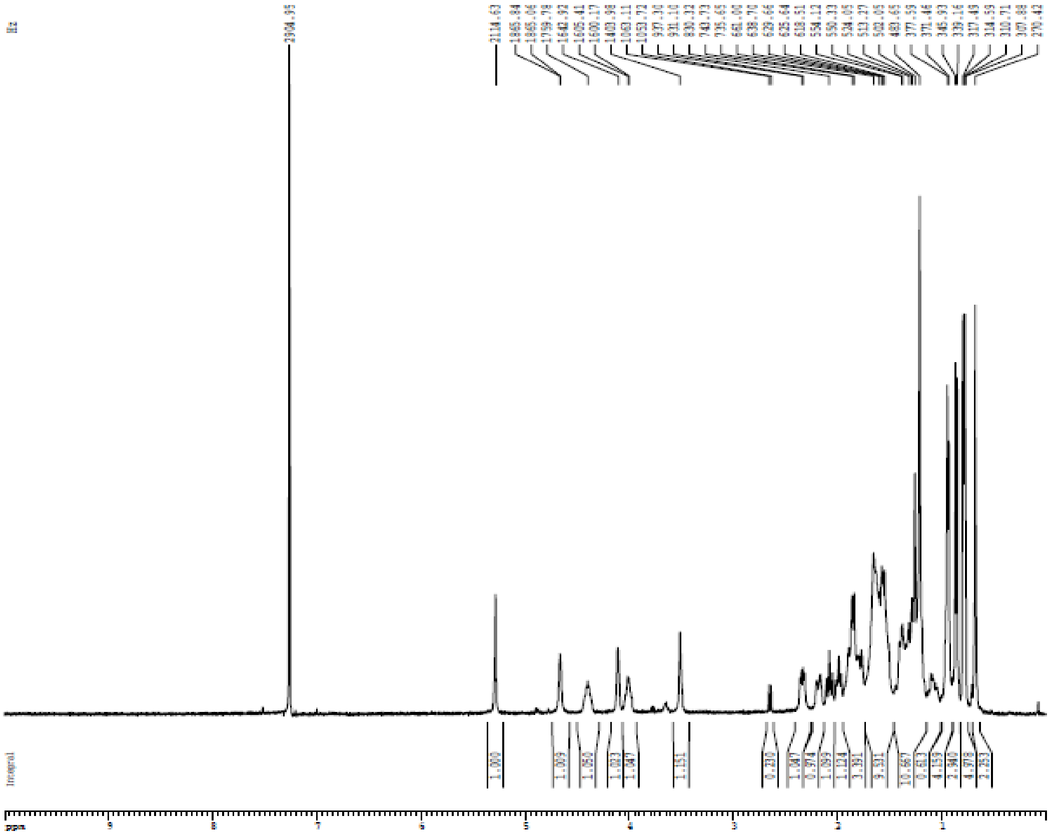

.

Figure S19. ^{13}C MNR (125 MHz, $CDCl_3$) spectrum of the new compound **3**.

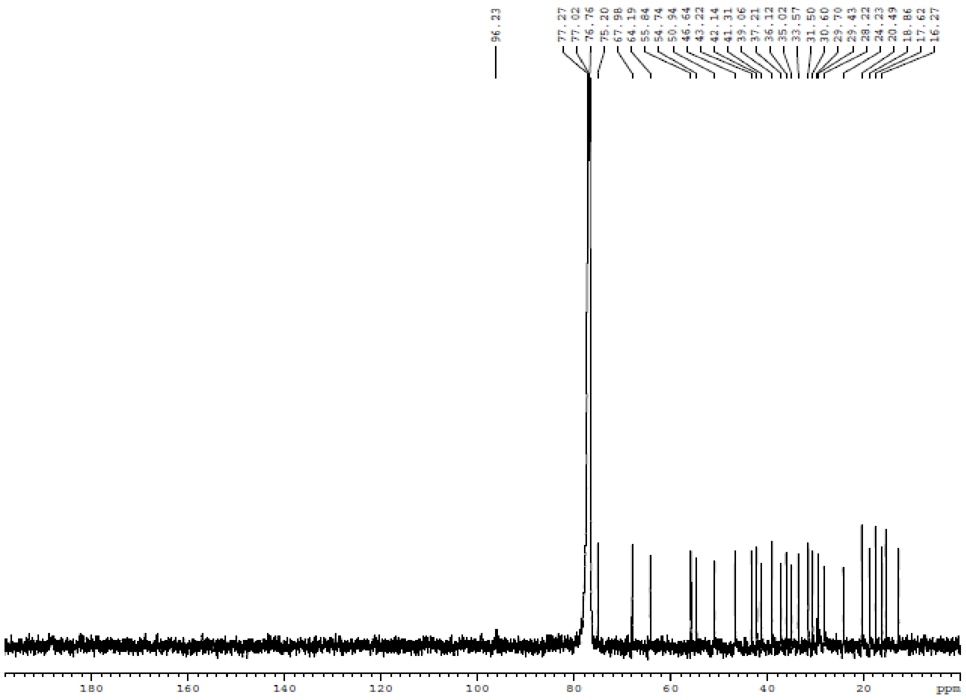

Figure S20. DEPT (125 MHz, $CDCl_3$) spectrum of the new compound **3**.

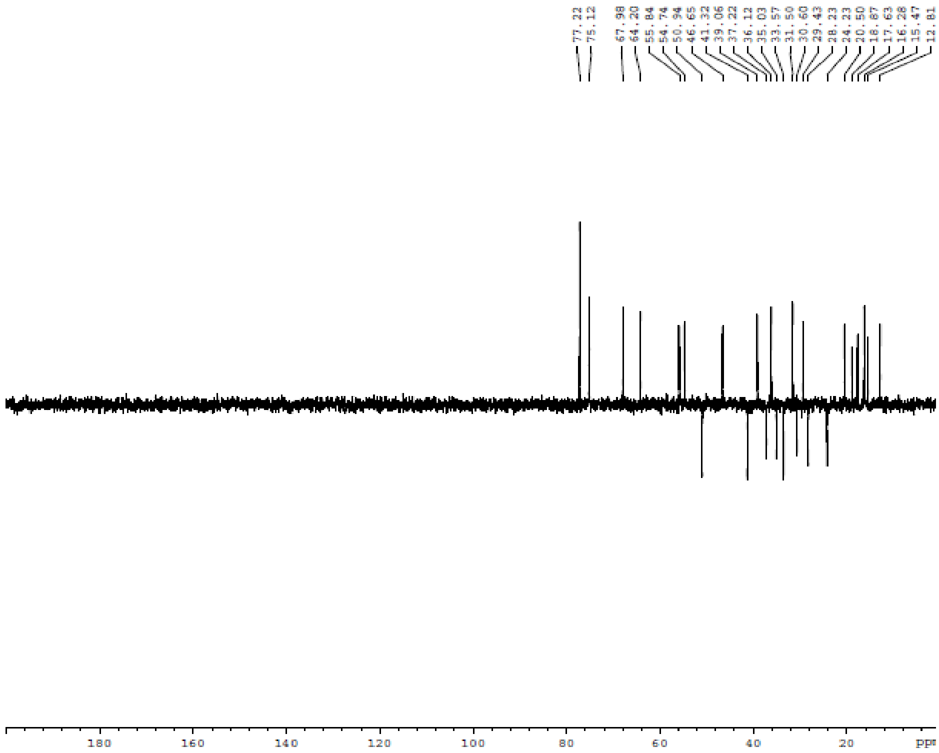

Figure S21. HSQC spectrum of the new compound **3**.

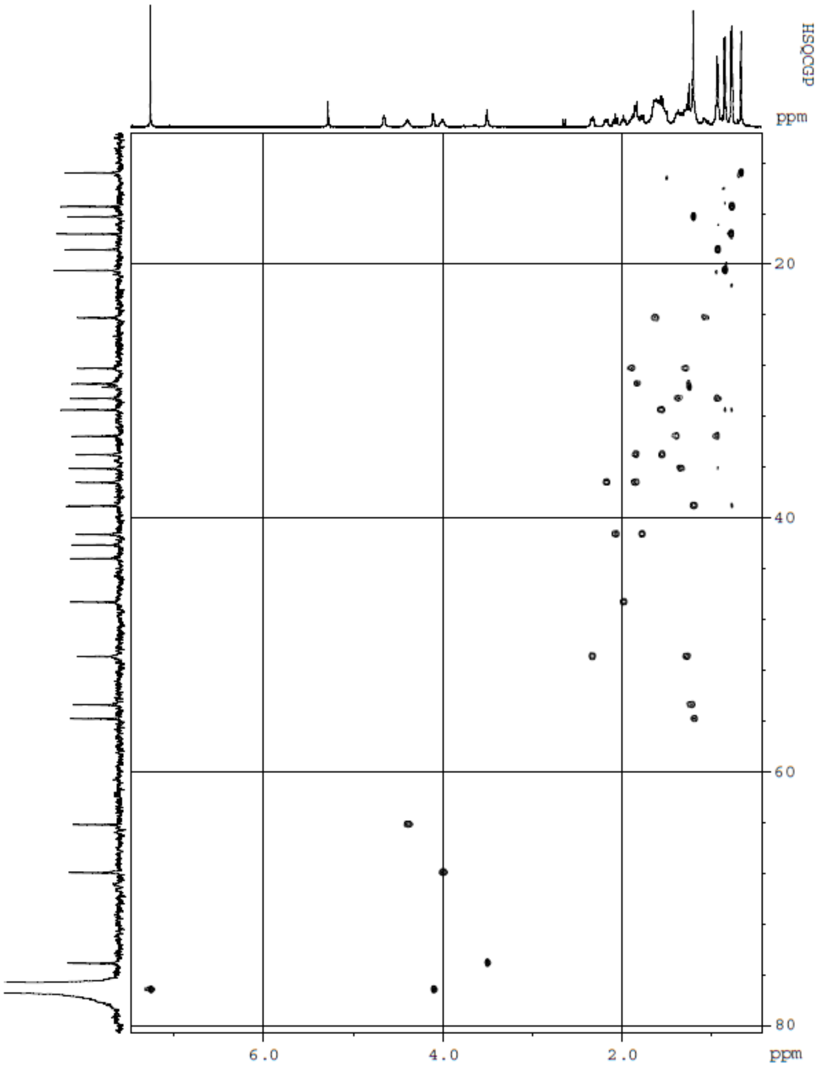

Figure S22. ^{1}H-^{1}H COSY spectrum of the new compound **3**.

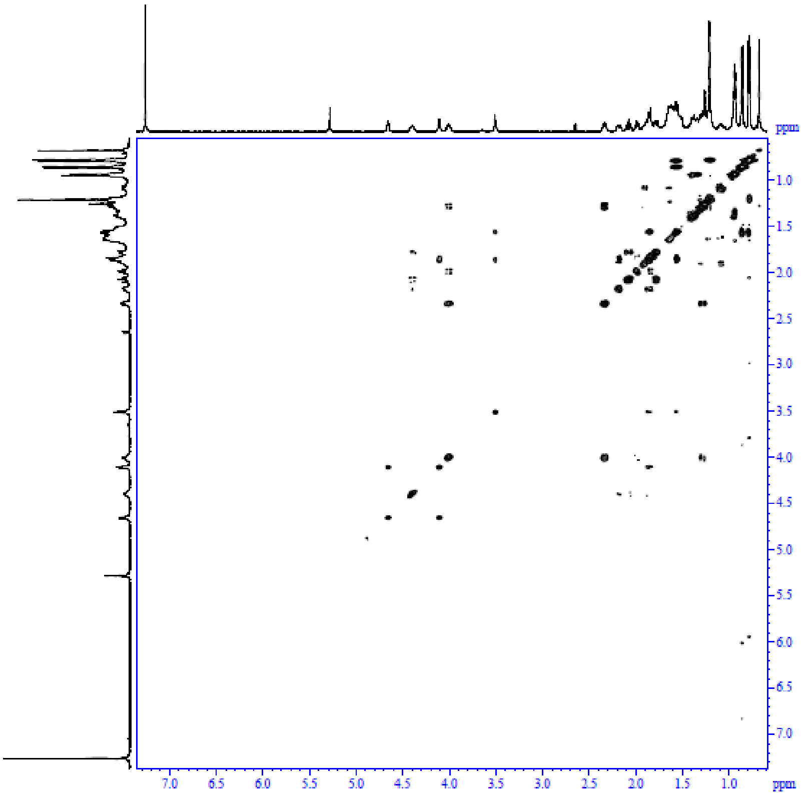

Figure S23. HMBC spectrum of the new compound **3**.

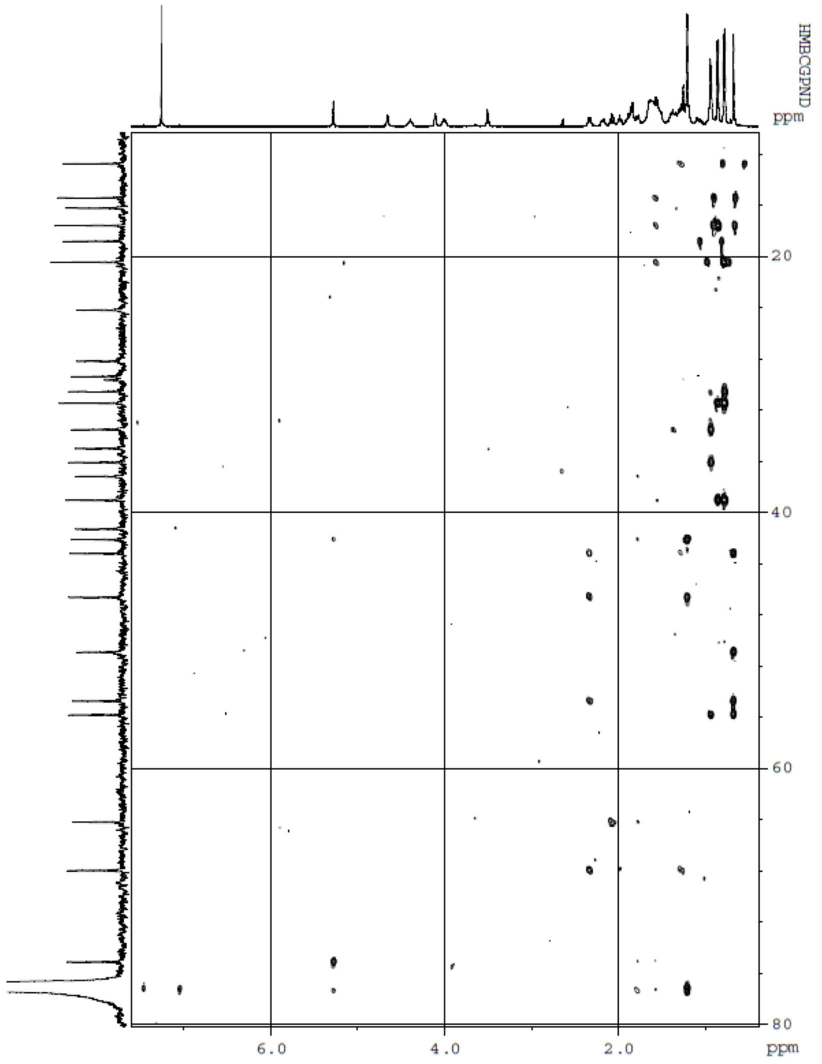

Figure S24. NOESY spectrum of the new compound **3**.

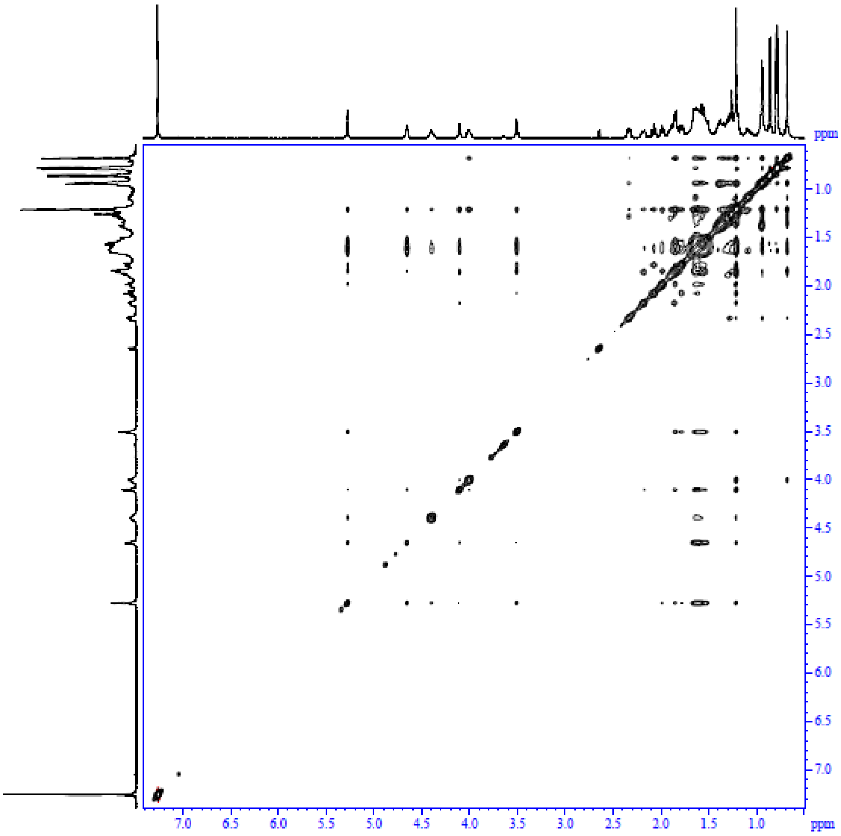

Figure S25. HR-ESIMS spectrum of the new compound **4**.

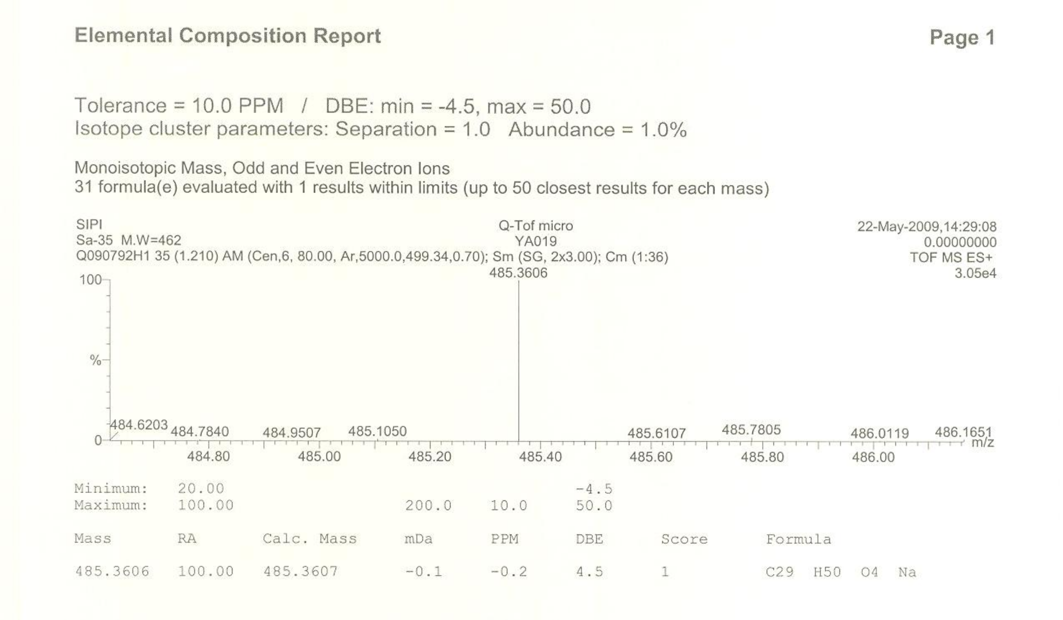

Mass	RA	Calc. Mass	mDa	PPM	DBE	Score	Formula
485.3606	100.00	485.3607	-0.1	-0.2	4.5	1	C29 H50 O4 Na

Figure S26. ^{1}H MNR (400 MHz, $CDCl_3$) spectrum of the new compound **4**.

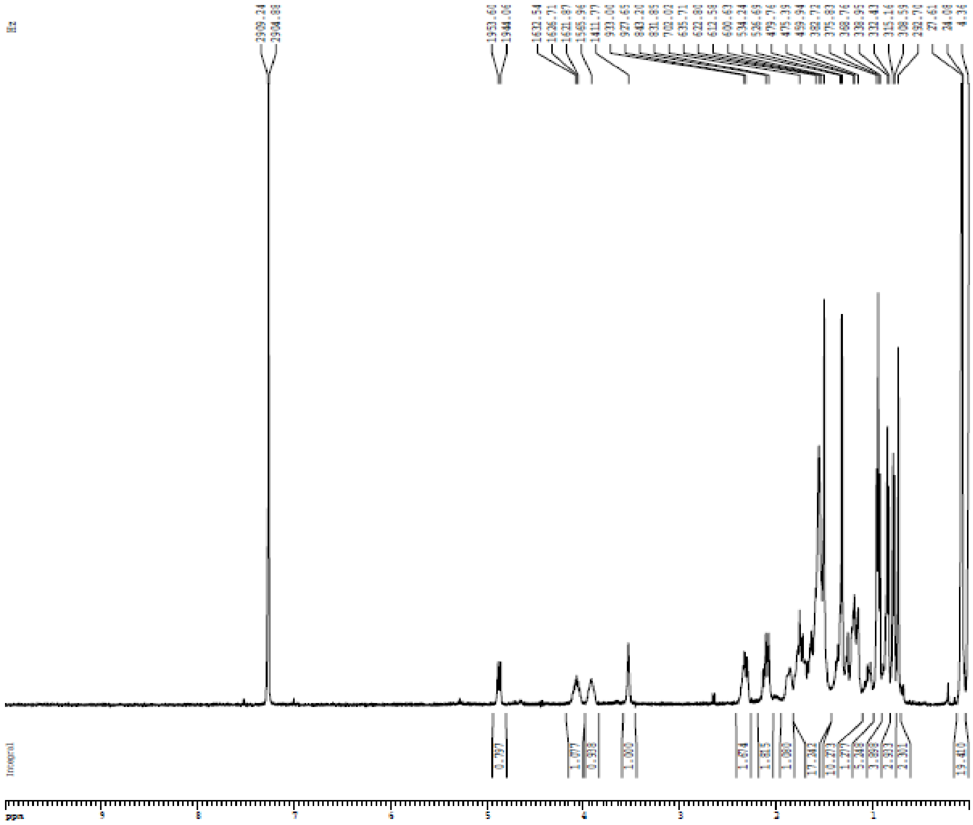

Figure S27. ^{13}C MNR (100 MHz, $CDCl_3$) spectrum of the new compound **4**.

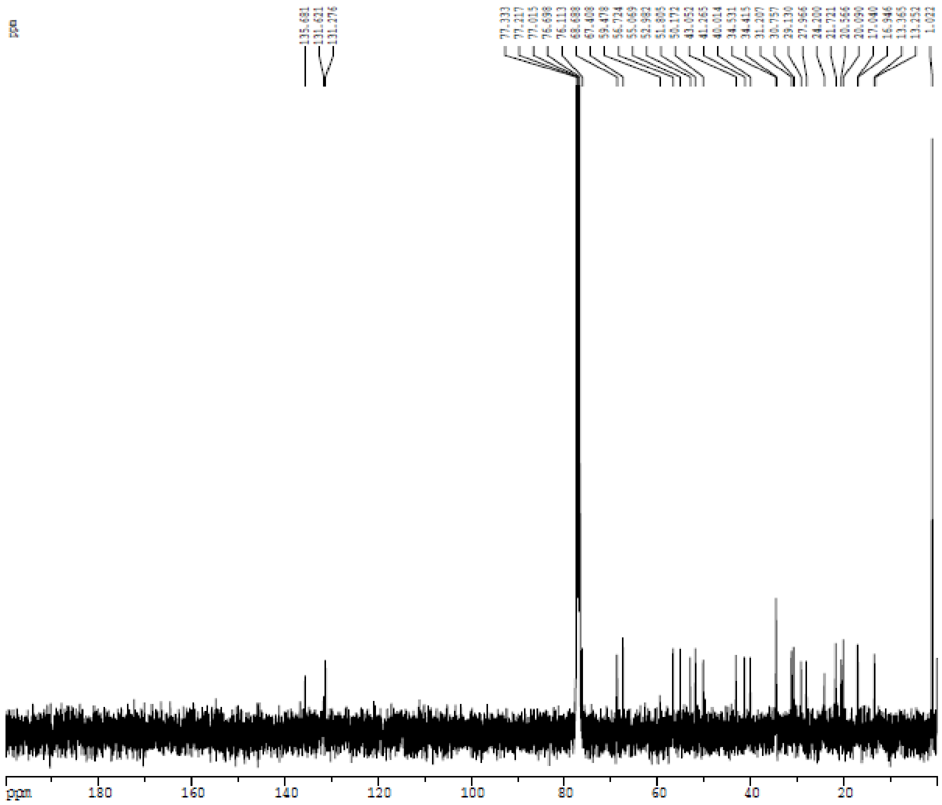

Figure S28. DEPT (100 MHz, $CDCl_3$) spectrum of the new compound **4**.

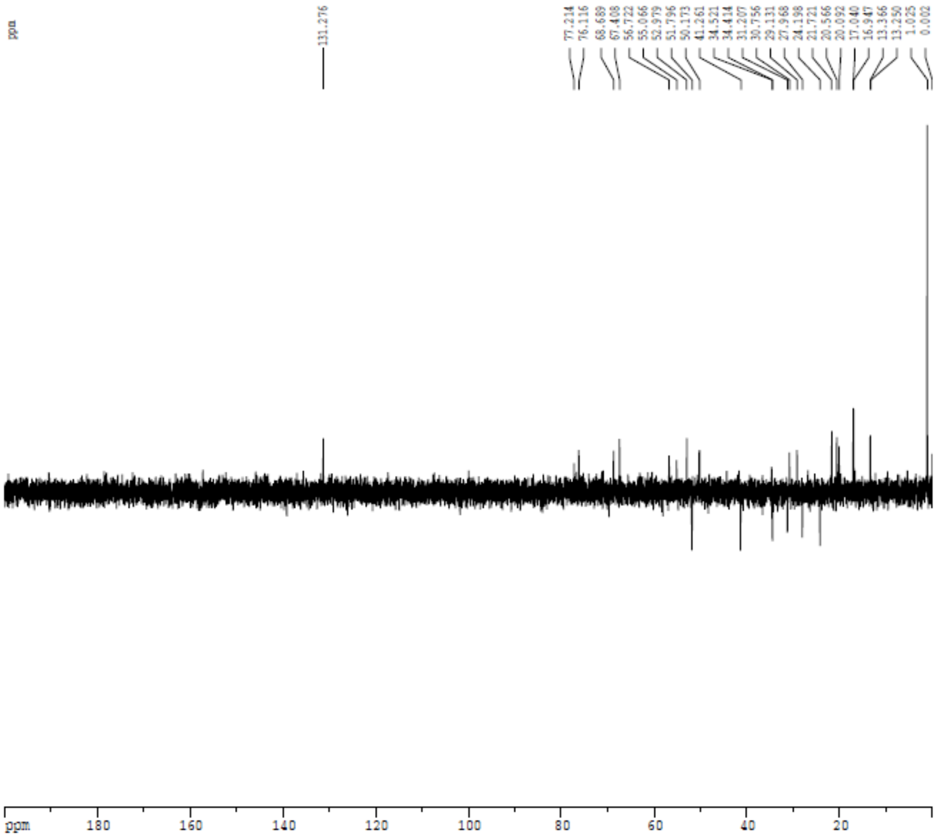

Figure S29. HSQC spectrum of the new compound **4**.

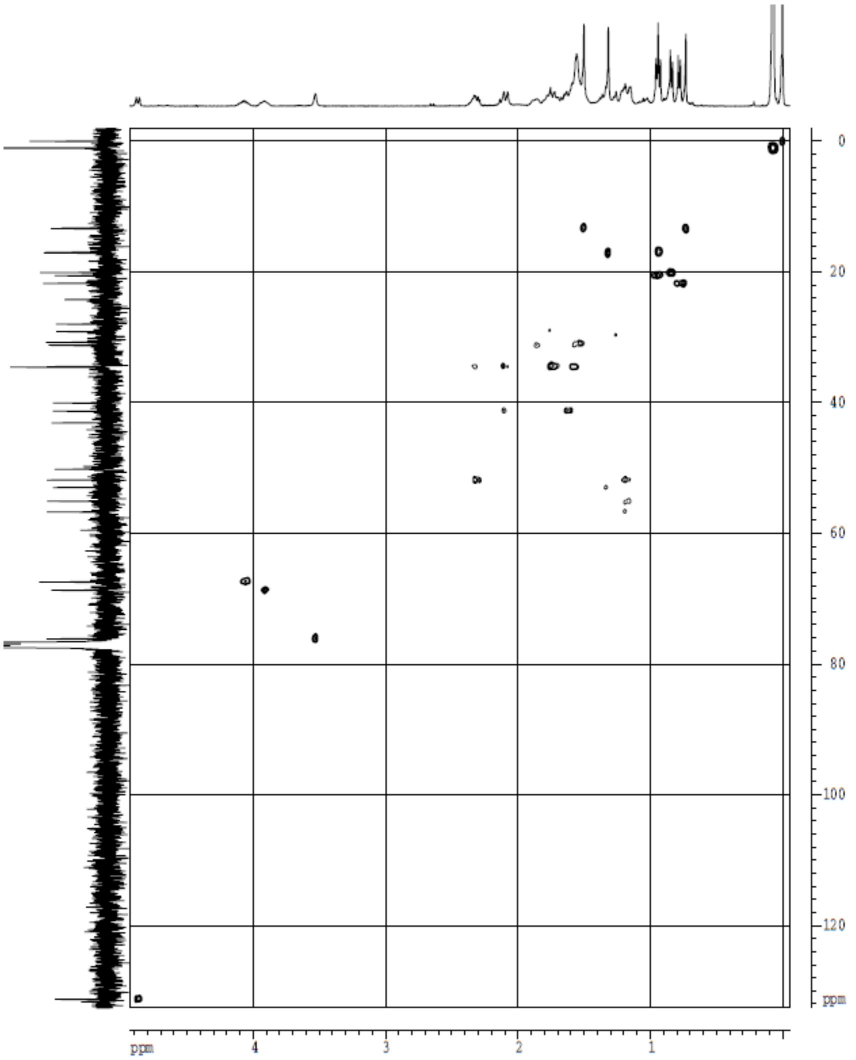

Figure S30. ^{1}H-^{1}H COSY spectrum of the new compound **4**.

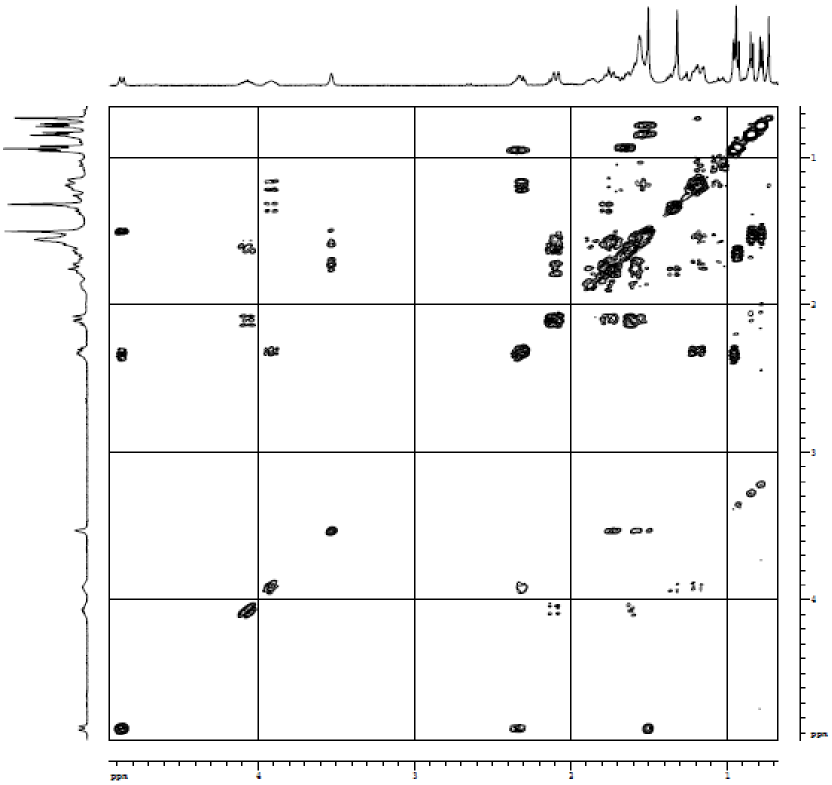

Figure S31. HMBC spectrum of the new compound **4**.

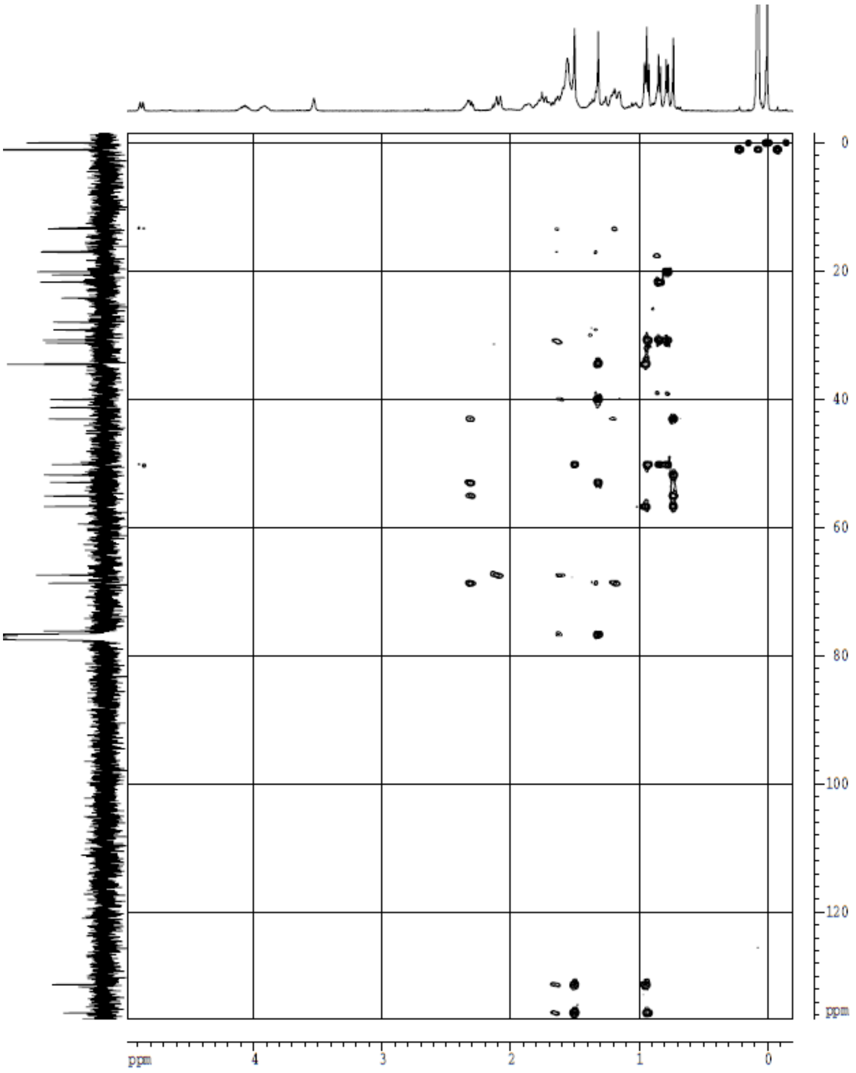

Figure S32. NOESY spectrum of the new compound **4**.

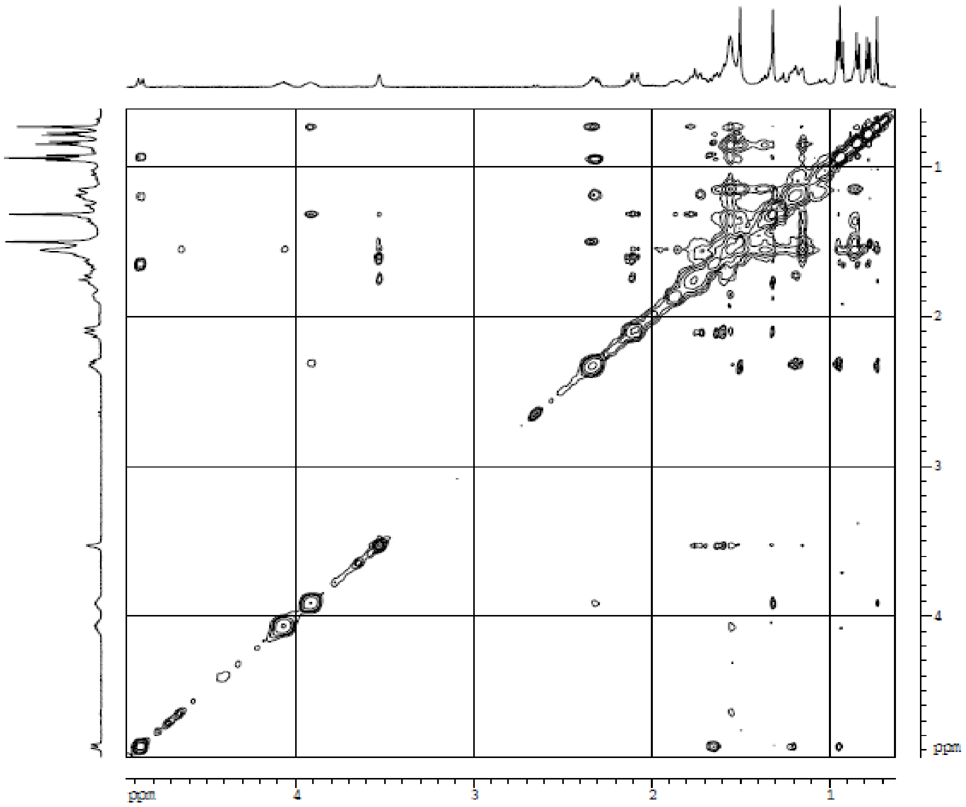

Figure S33. HR-ESIMS spectrum of the new compound **5**.

Elemental Composition Report **Page 1**

Multiple Mass Analysis: 2 mass(es) processed - displaying only valid results
Tolerance = 10.0 PPM / DBE: min = -4.5, max = 50.0
Isotope cluster parameters: Separation = 1.0 Abundance = 1.0%

Monoisotopic Mass, Odd and Even Electron Ions
38 formula(e) evaluated with 2 results within limits (up to 50 closest results for each mass)

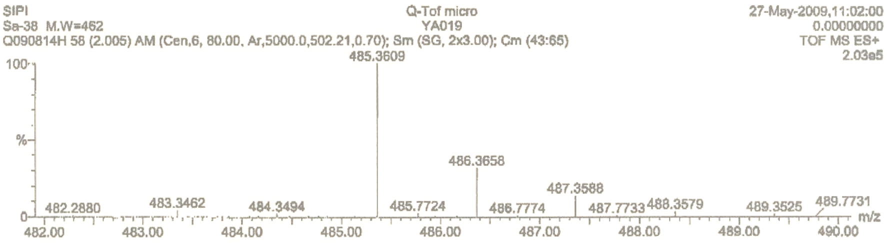

Minimum:	15.00				-4.5		
Maximum:	100.00		200.0	10.0	50.0		
Mass	RA	Calc. Mass	mDa	PPM	DBE	Score	Formula
485.3609	100.00	485.3607	0.2	0.5	4.5	1	C29 H50 O4 Na
486.3658	31.15	486.3685	-2.7	-5.6	4.0	1	C29 H51 O4 Na

Figure S34. ^{1}H MNR (400 MHz, $CDCl_3$) spectrum of the new compound **5**.

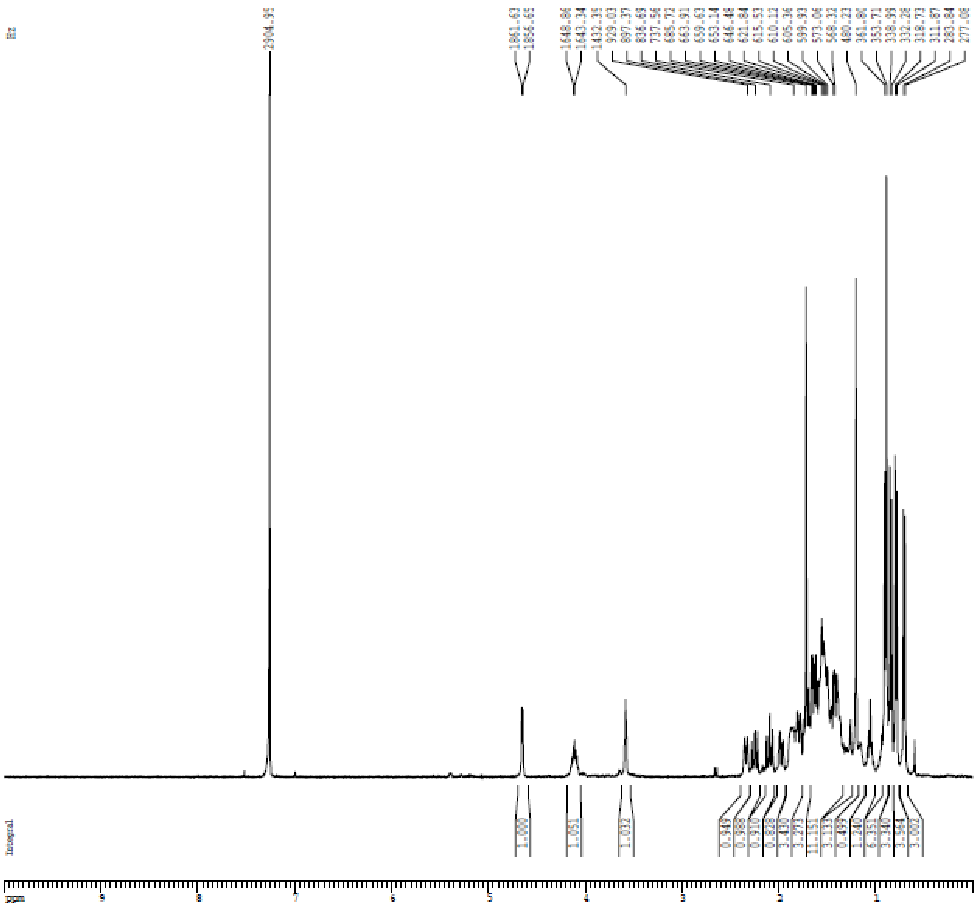

Figure S35. ^{13}C MNR (100 MHz, $CDCl_3$) spectrum of the new compound **5**.

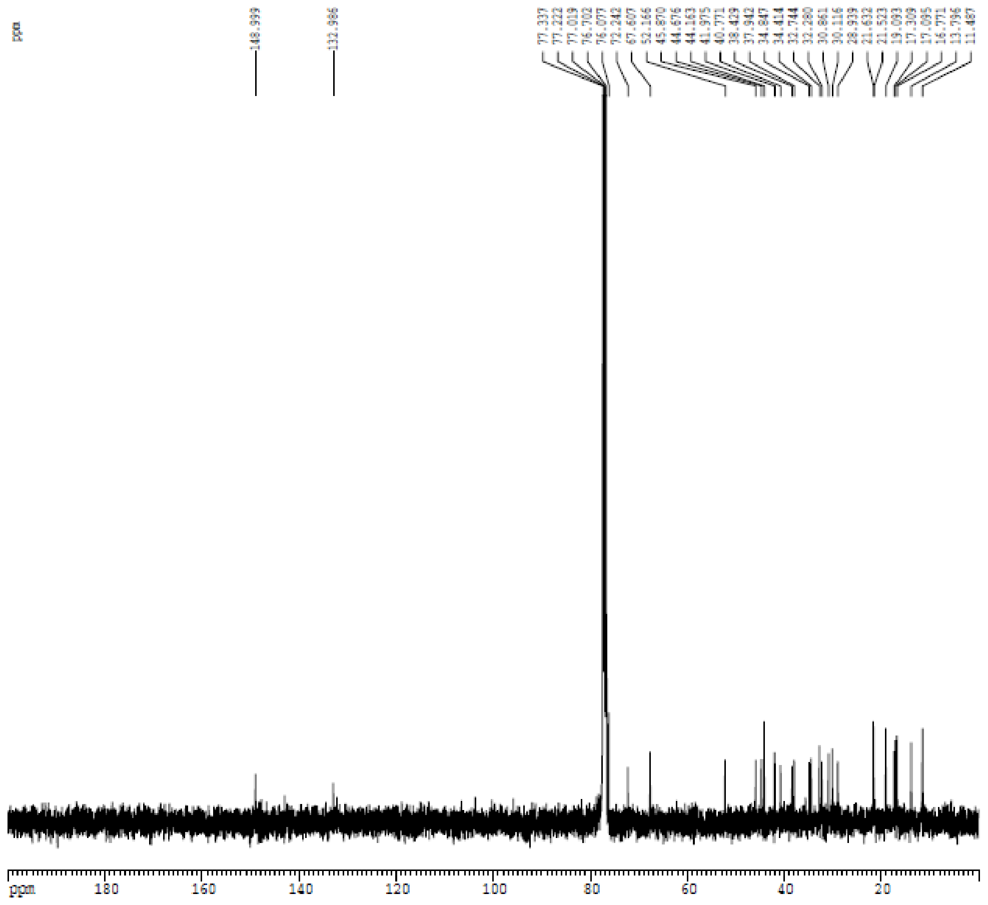

Figure S36. DEPT (100 MHz, $CDCl_3$) spectrum (1) of the new compound **5**.

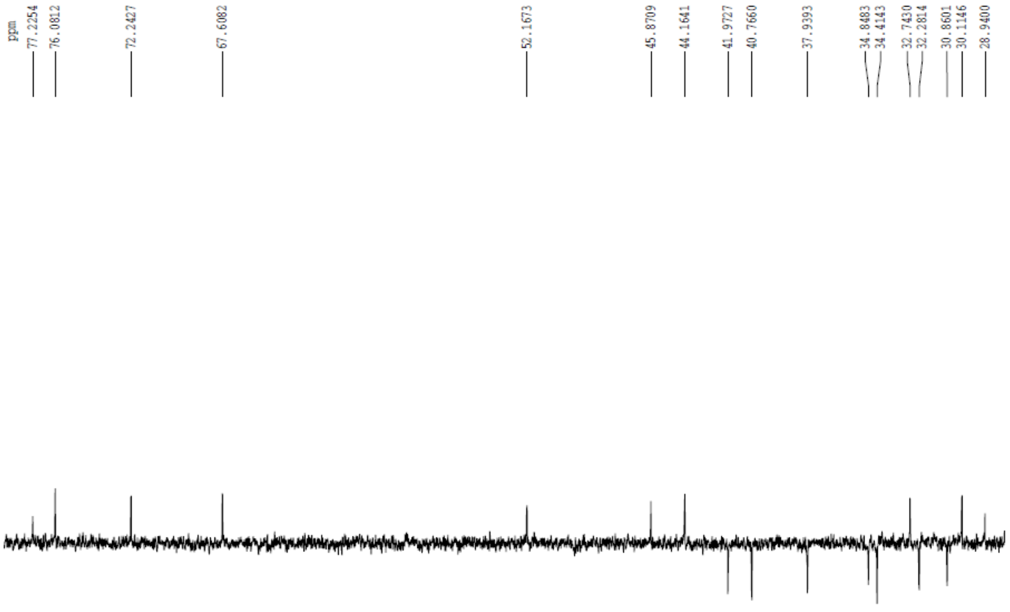

Figure S37. DEPT (100 MHz, $CDCl_3$) spectrum (2) of the new compound **5**.

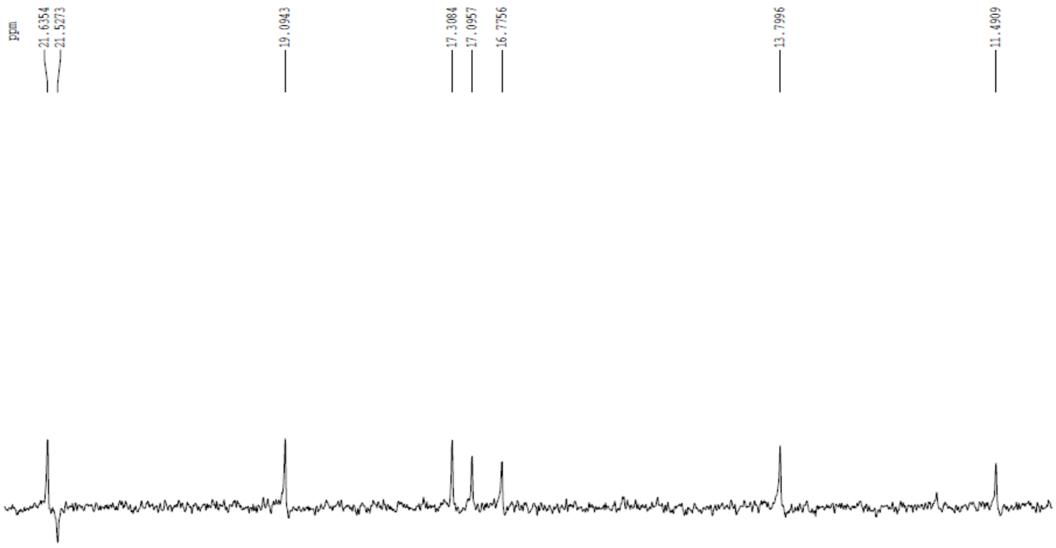

Figure S38. ^{1}H-^{1}H COSY spectrum of the new compound **5**.

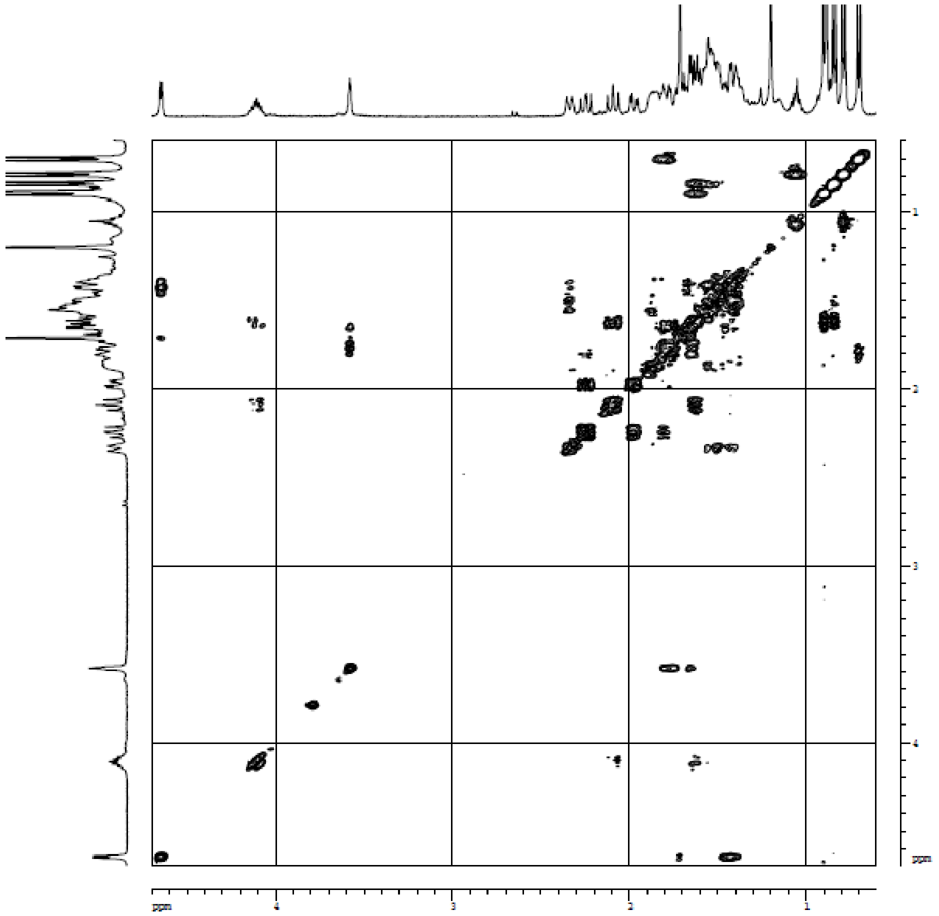

Figure S39. HSQC spectrum of the new compound **5**.

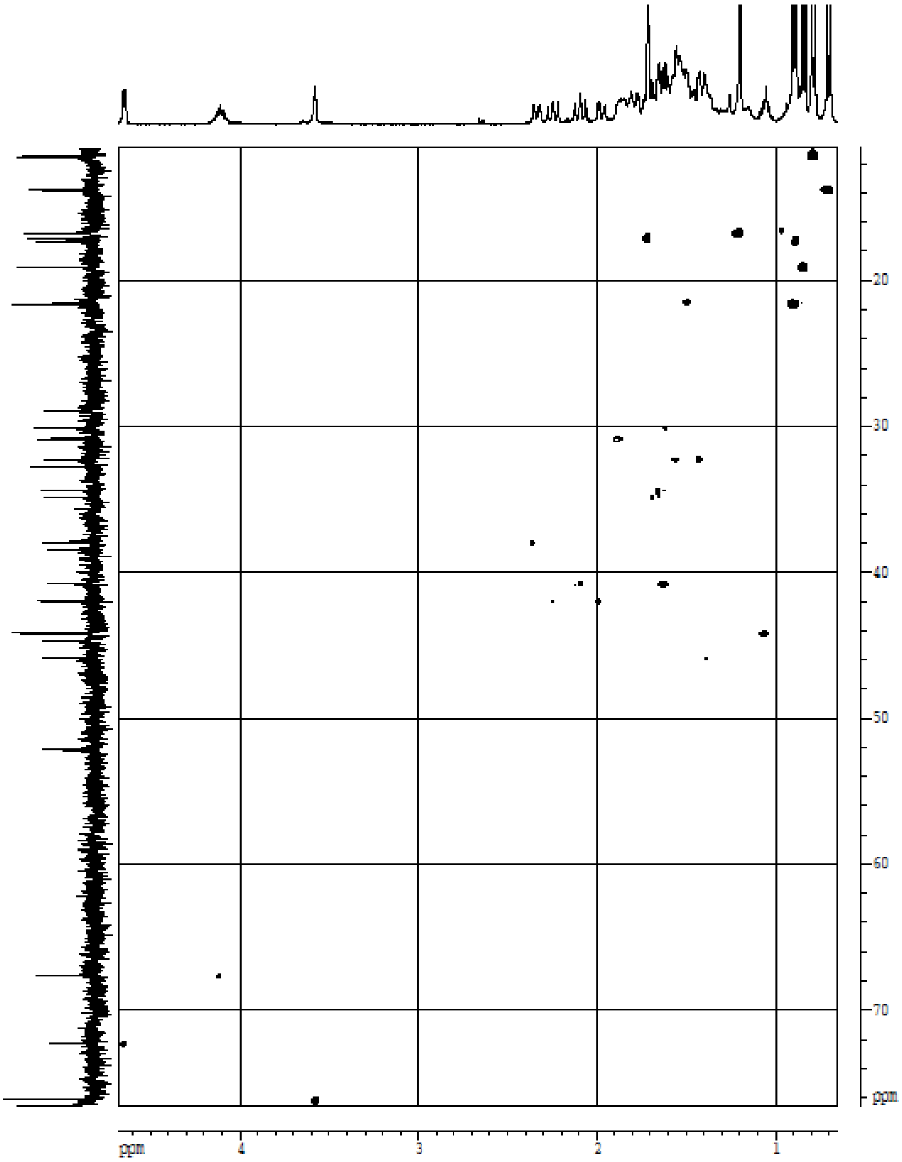

Figure S40. HMBC spectrum of the new compound **5**.

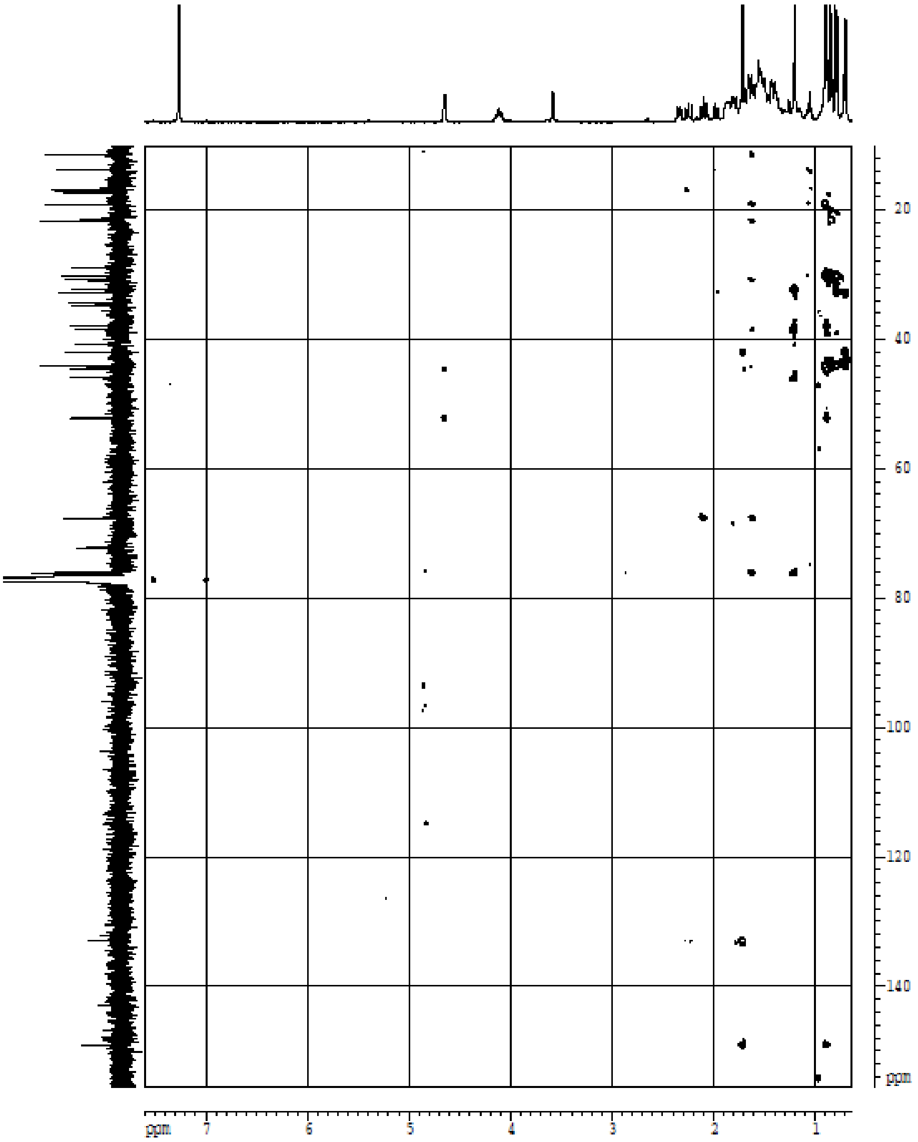

Figure S41. NOESY spectrum of the new compound **5**.

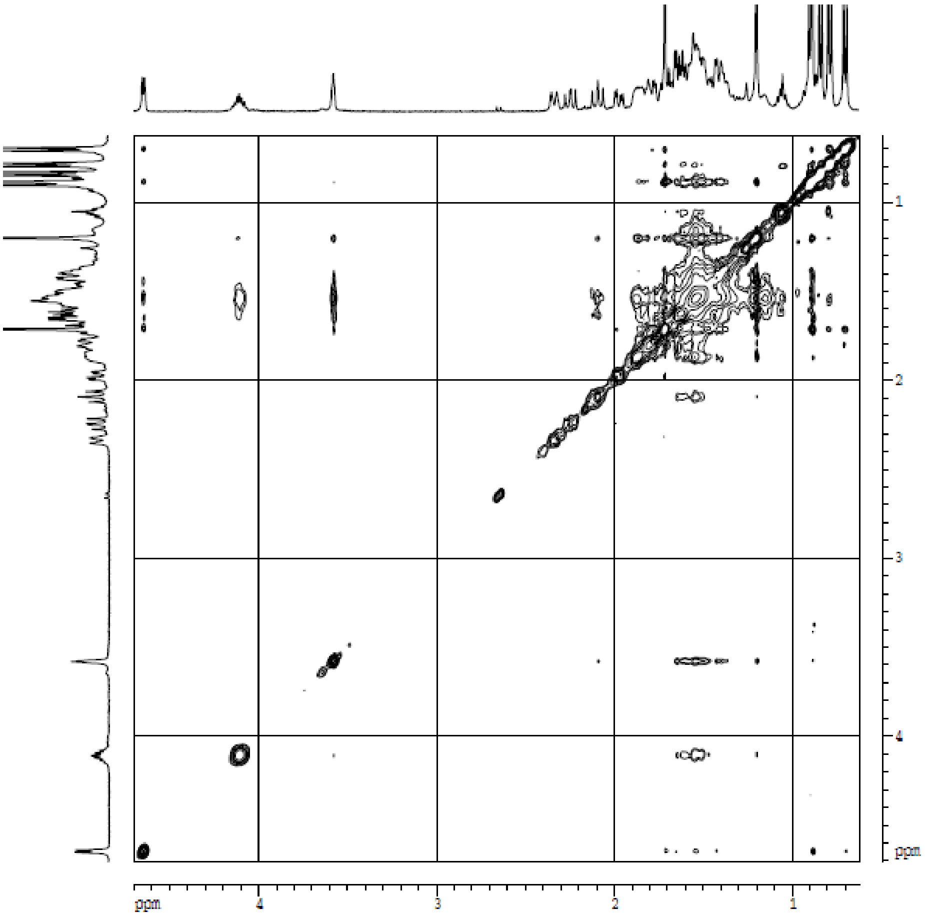

Figure S42. HR-ESIMS spectrum of the new compound **6**.

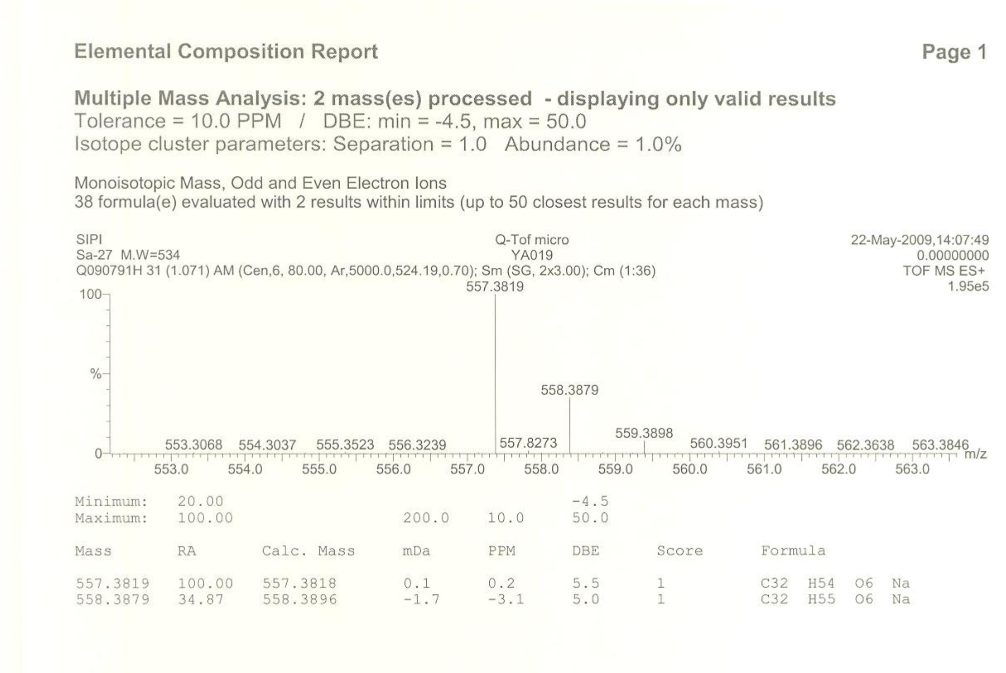
Elemental Composition Report Page 1

Multiple Mass Analysis: 2 mass(es) processed - displaying only valid results
Tolerance = 10.0 PPM / DBE: min = -4.5, max = 50.0
Isotope cluster parameters: Separation = 1.0 Abundance = 1.0%

Monoisotopic Mass, Odd and Even Electron Ions
38 formula(e) evaluated with 2 results within limits (up to 50 closest results for each mass)

SIPI Q-Tof micro 22-May-2009,14:07:49
Sa-27 M.W=534 YA019 0.00000000
Q090791H 31 (1.071) AM (Cen,6, 80.00, Ar,5000.0,524.19,0.70); Sm (SG, 2x3.00); Cm (1:36) TOF MS ES+ 1.95e5

Minimum:	20.00				-4.5		
Maximum:	100.00		200.0	10.0	50.0		
Mass	RA	Calc. Mass	mDa	PPM	DBE	Score	Formula
557.3819	100.00	557.3818	0.1	0.2	5.5	1	C32 H54 O6 Na
558.3879	34.87	558.3896	-1.7	-3.1	5.0	1	C32 H55 O6 Na

Figure S43. ^{1}H MNR (400 MHz, $CDCl_3$) spectrum of the new compound **6**.

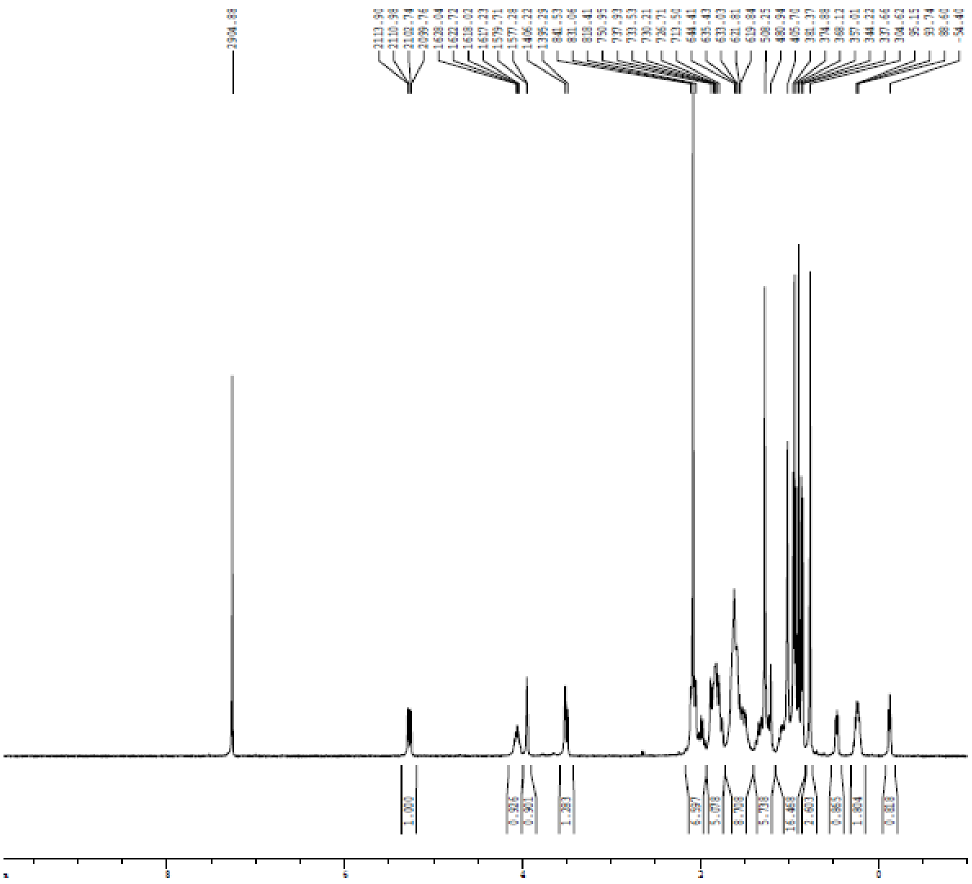

Figure S44. ^{13}C MNR (100 MHz, $CDCl_3$) spectrum of the new compound **6**

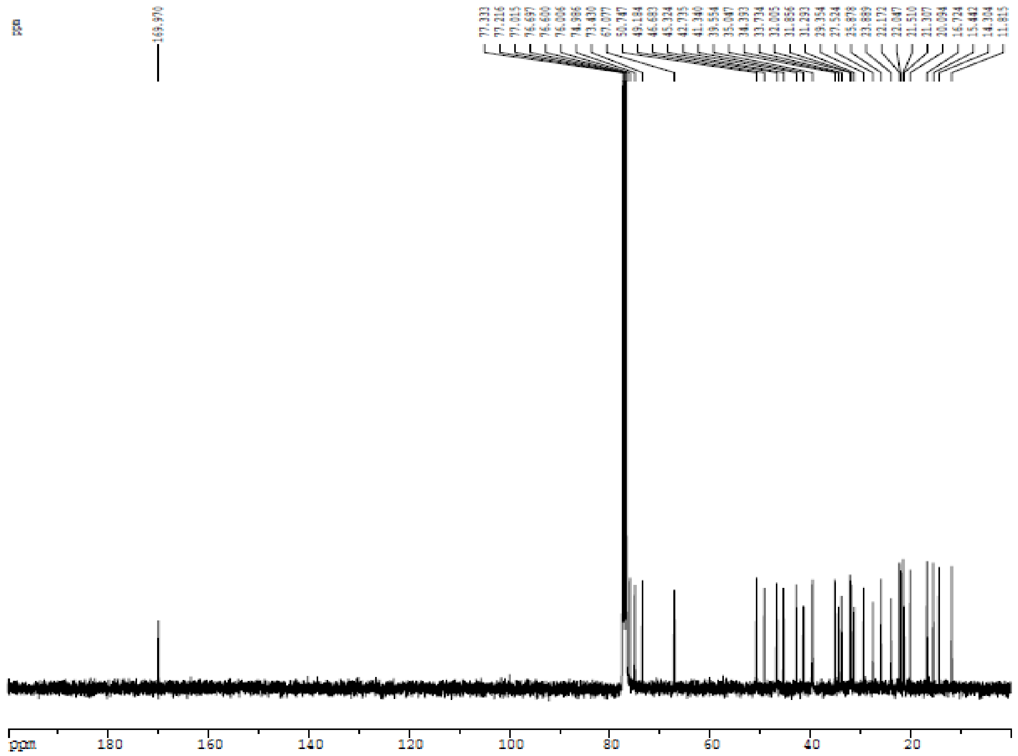

Figure S45. DEPT (100 MHz, $CDCl_3$) spectrum of the new compound **6**.

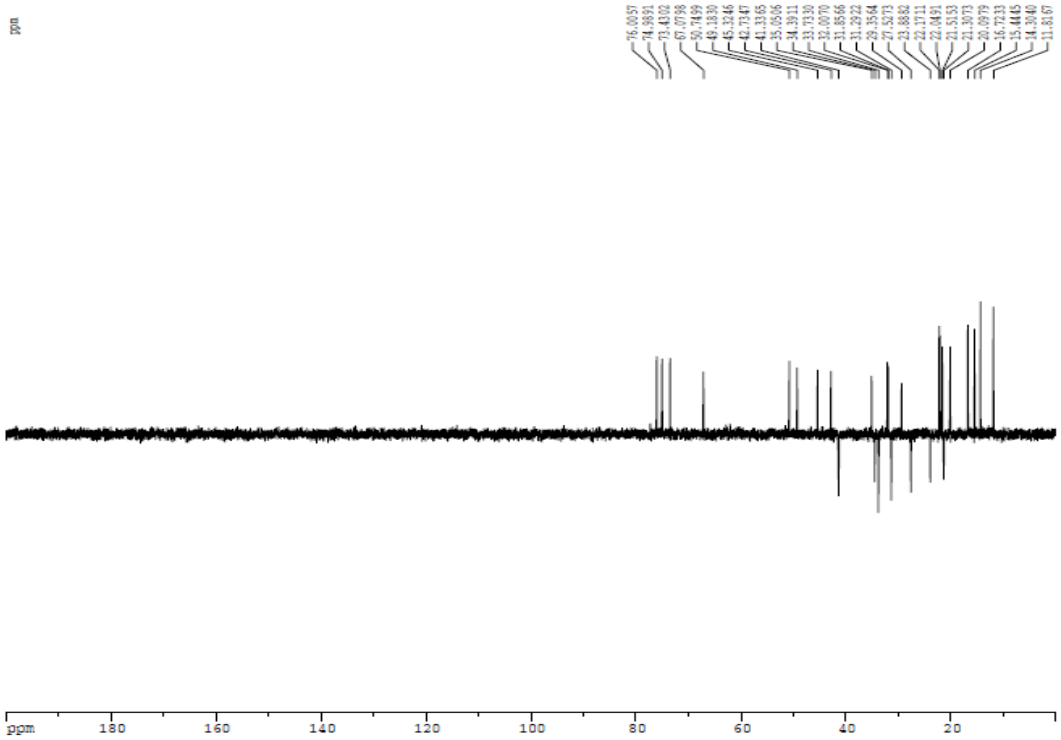

Figure S46. HSQC spectrum of the new compound **6**.

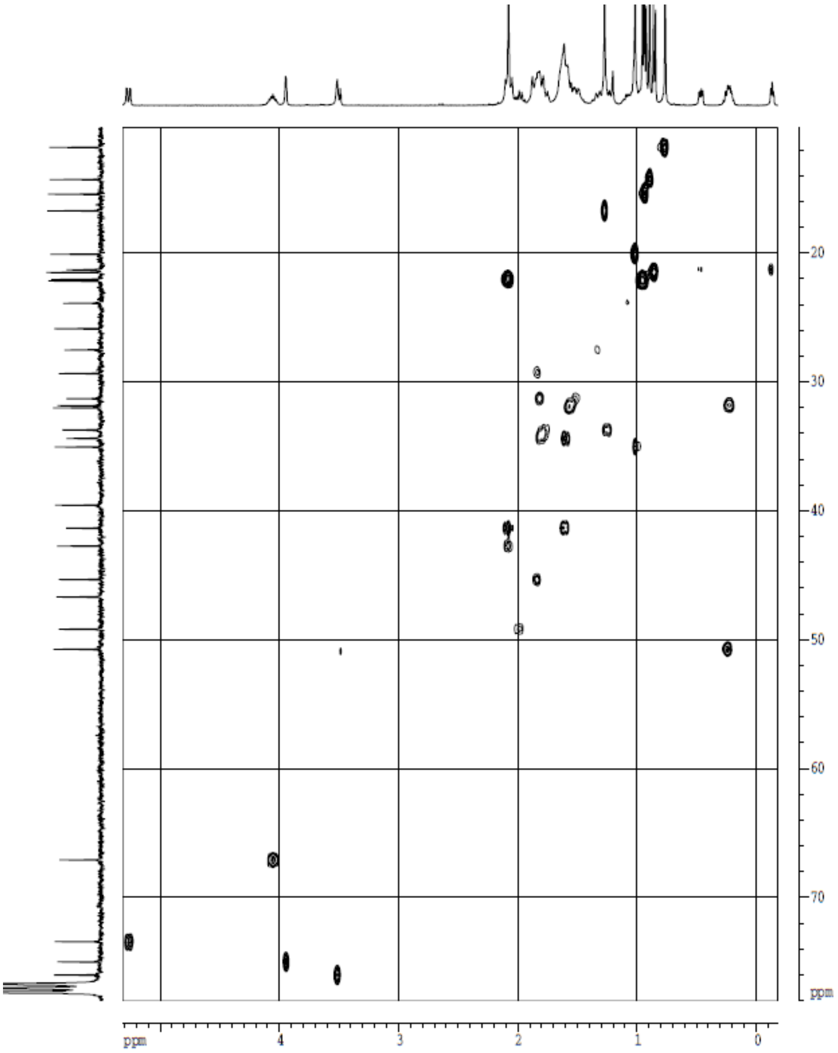

Figure S47. ^{1}H-^{1}H COSY spectrum of the new compound **6**.

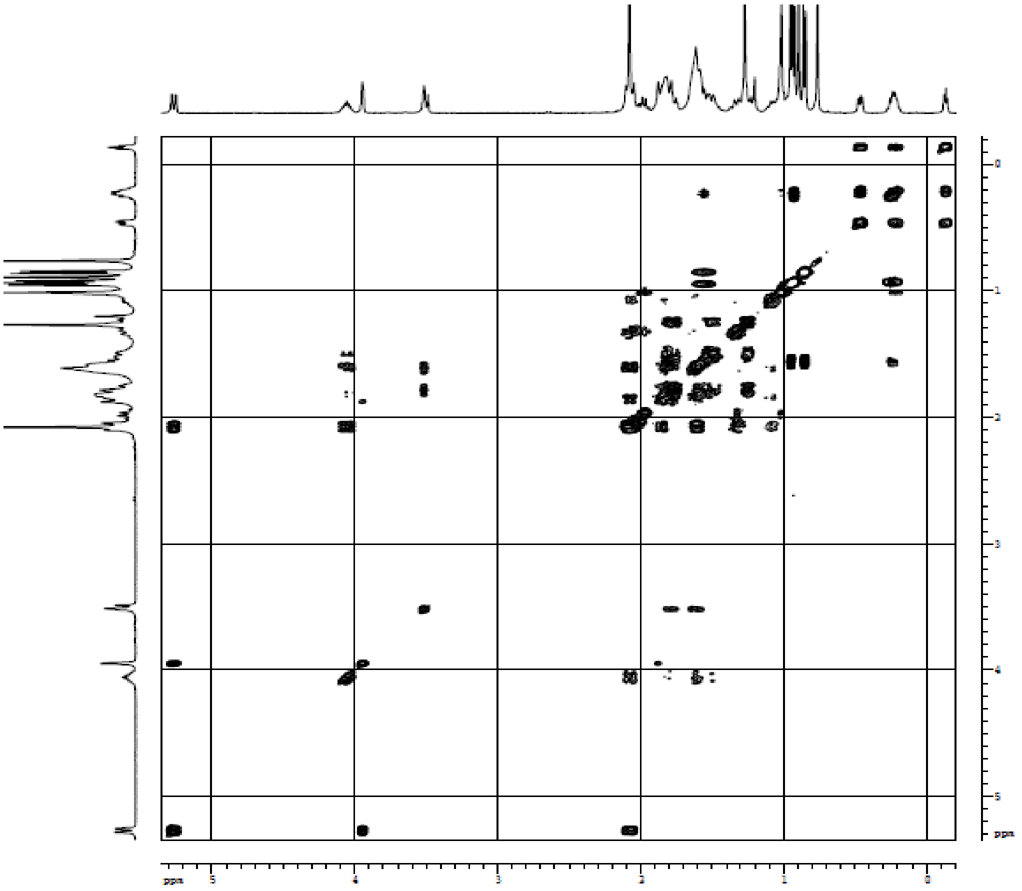

Figure S48. HMBC spectrum of the new compound **6**.

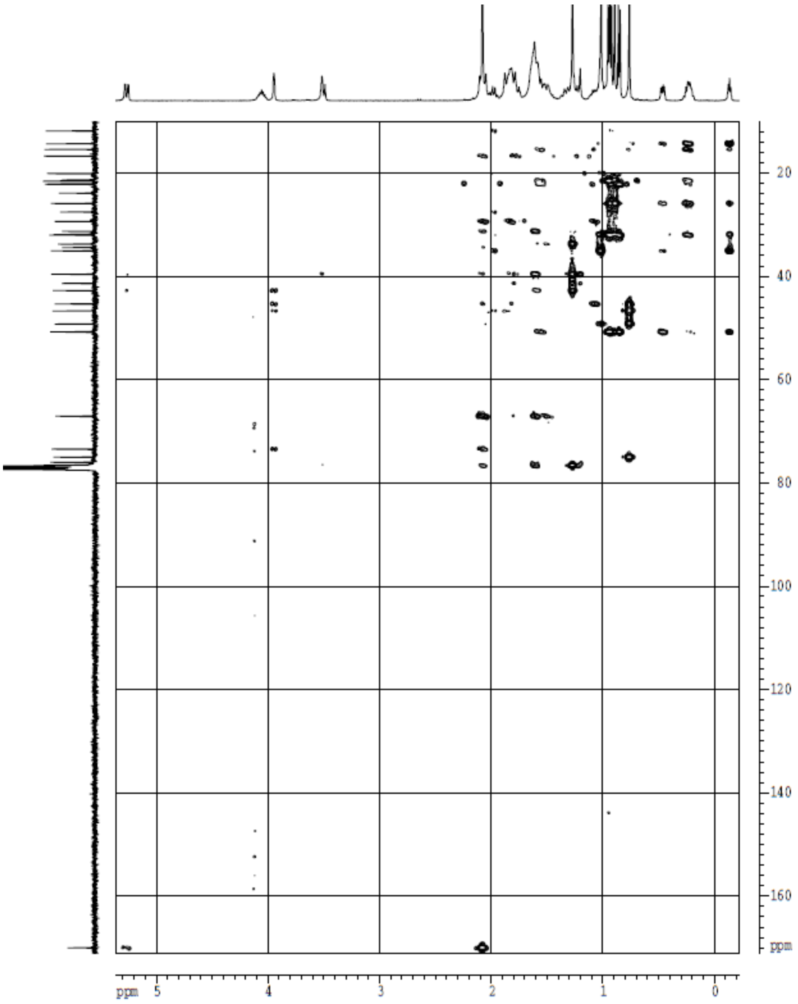

Figure S49. NOESY spectrum of the new compound **6**.

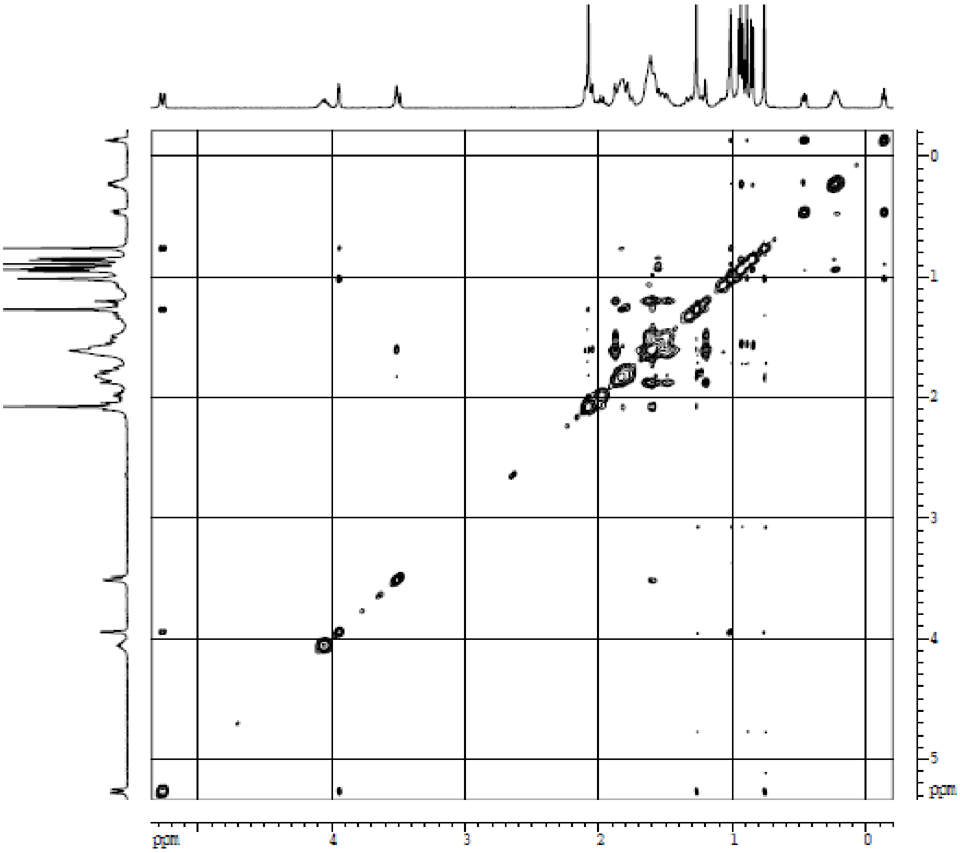

Figure S50. HR-ESIMS spectrum of the new compound **7**.

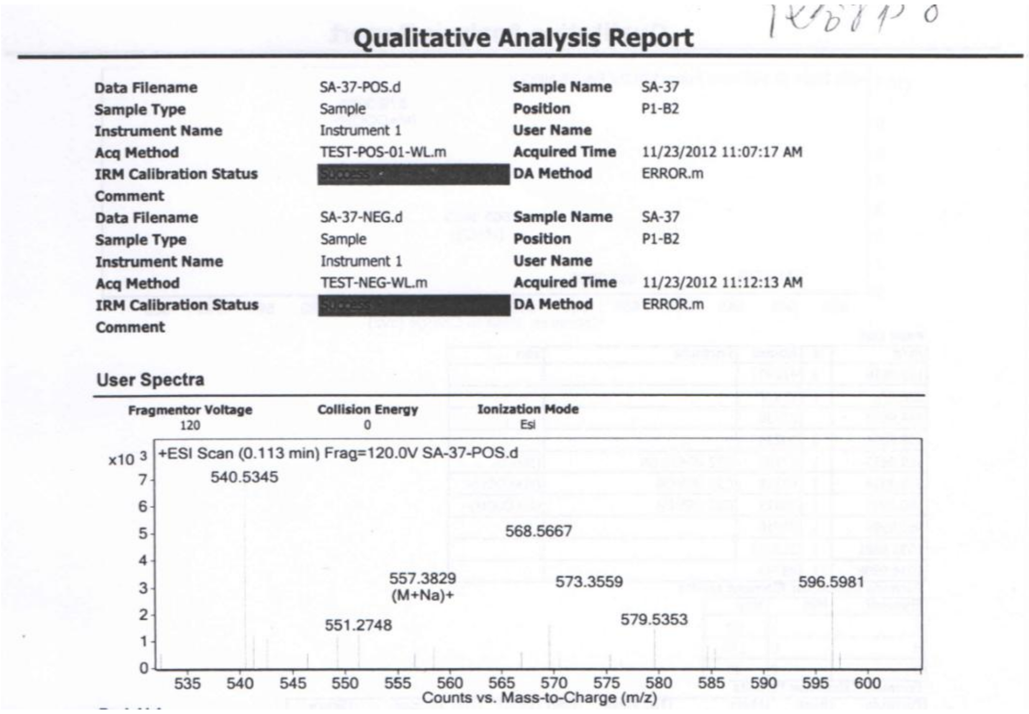

Figure S51. ^{1}H MNR (500 MHz, $CDCl_3$) spectrum of the new compound **7**.

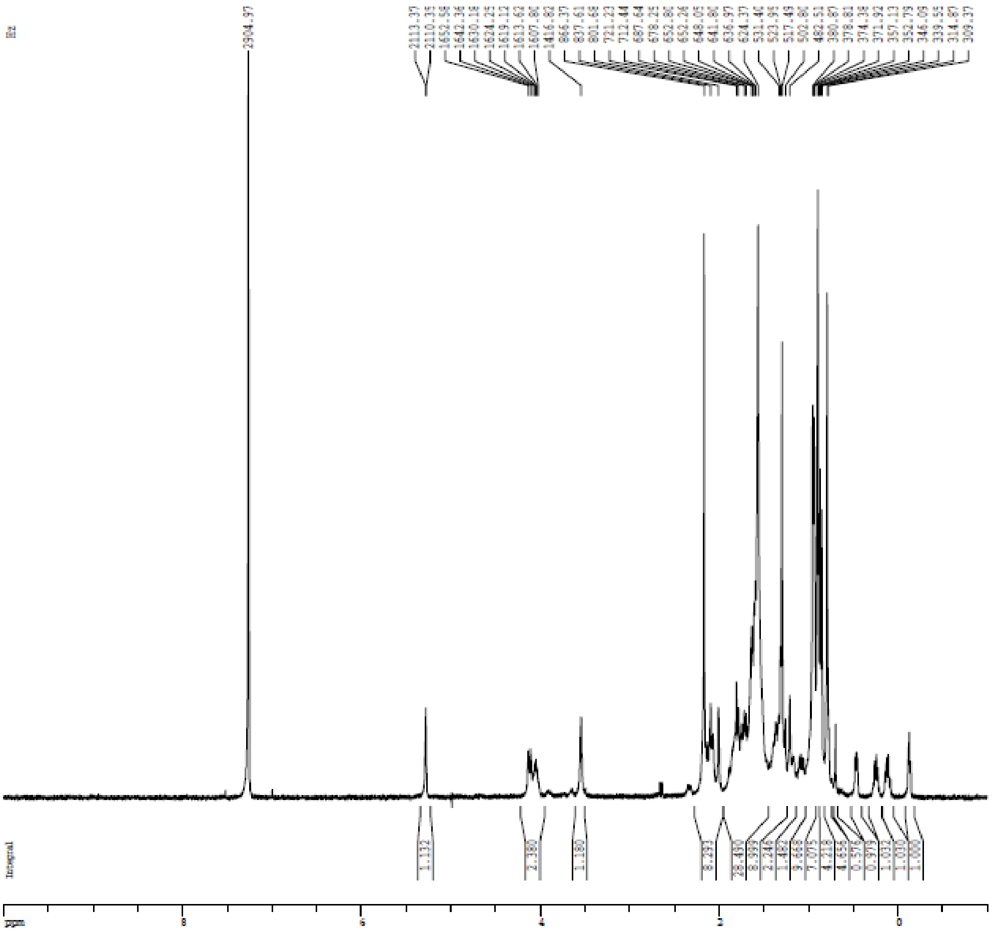

Figure S52. ^{13}C MNR (100 MHz, $CDCl_3$) spectrum of the new compound **7**.

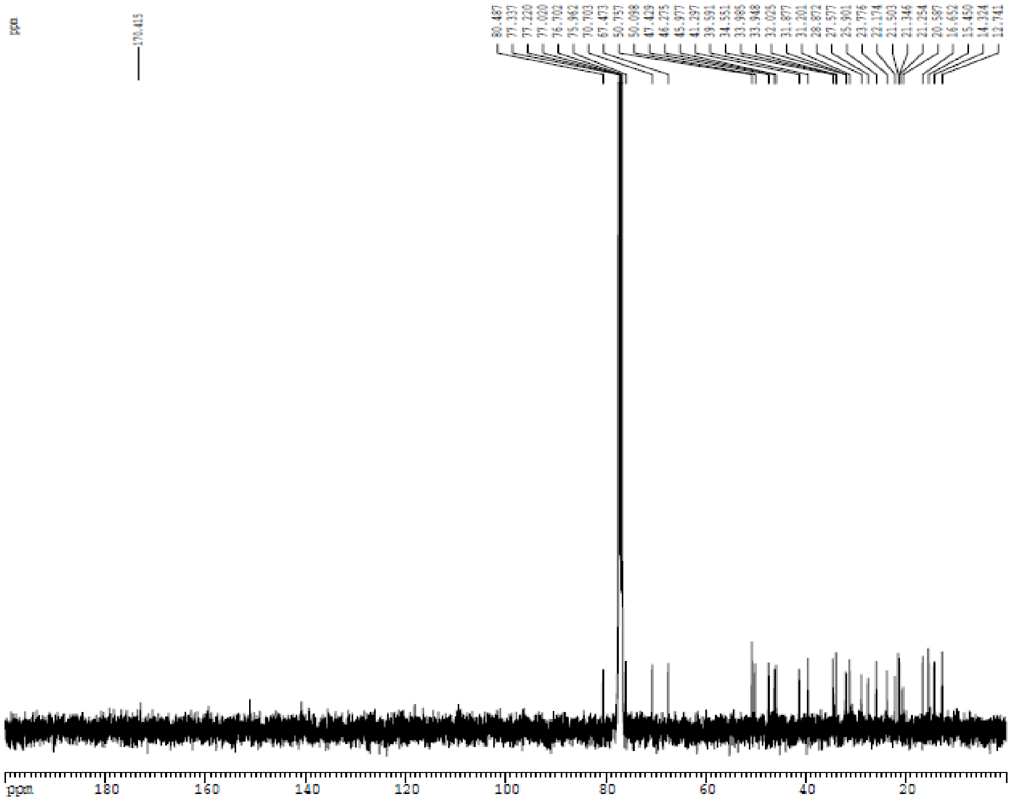

Figure S53. DEPT (100 MHz, $CDCl_3$) spectrum of the new compound **7**.

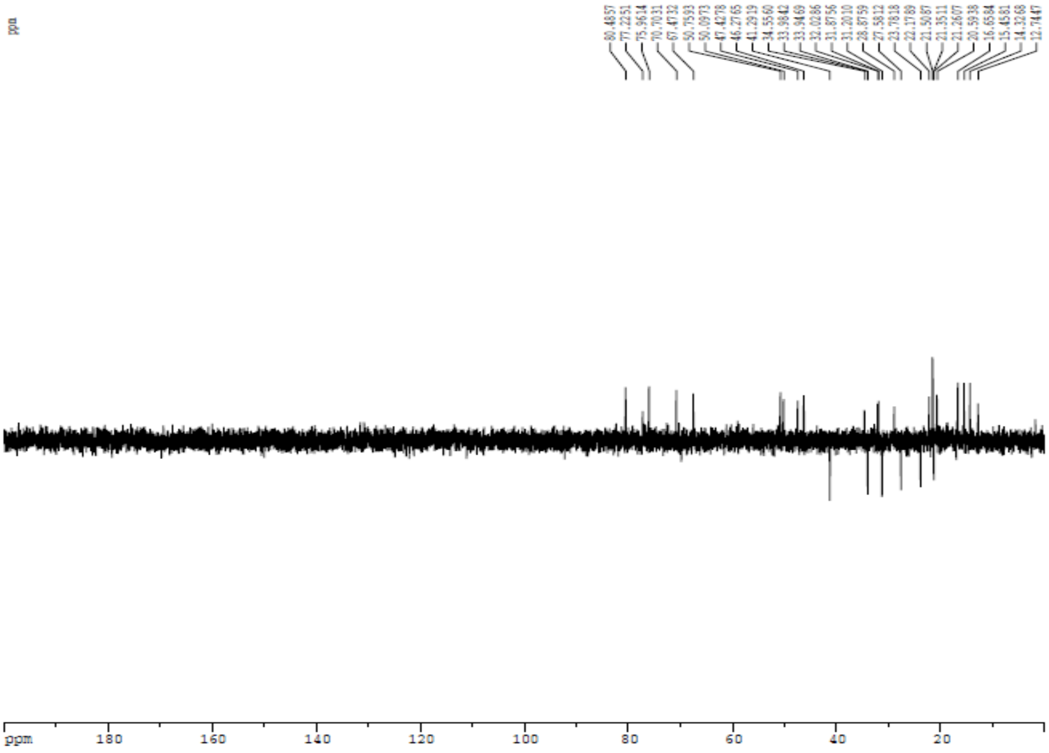

Figure S54. HSQC spectrum of the new compound **7**.

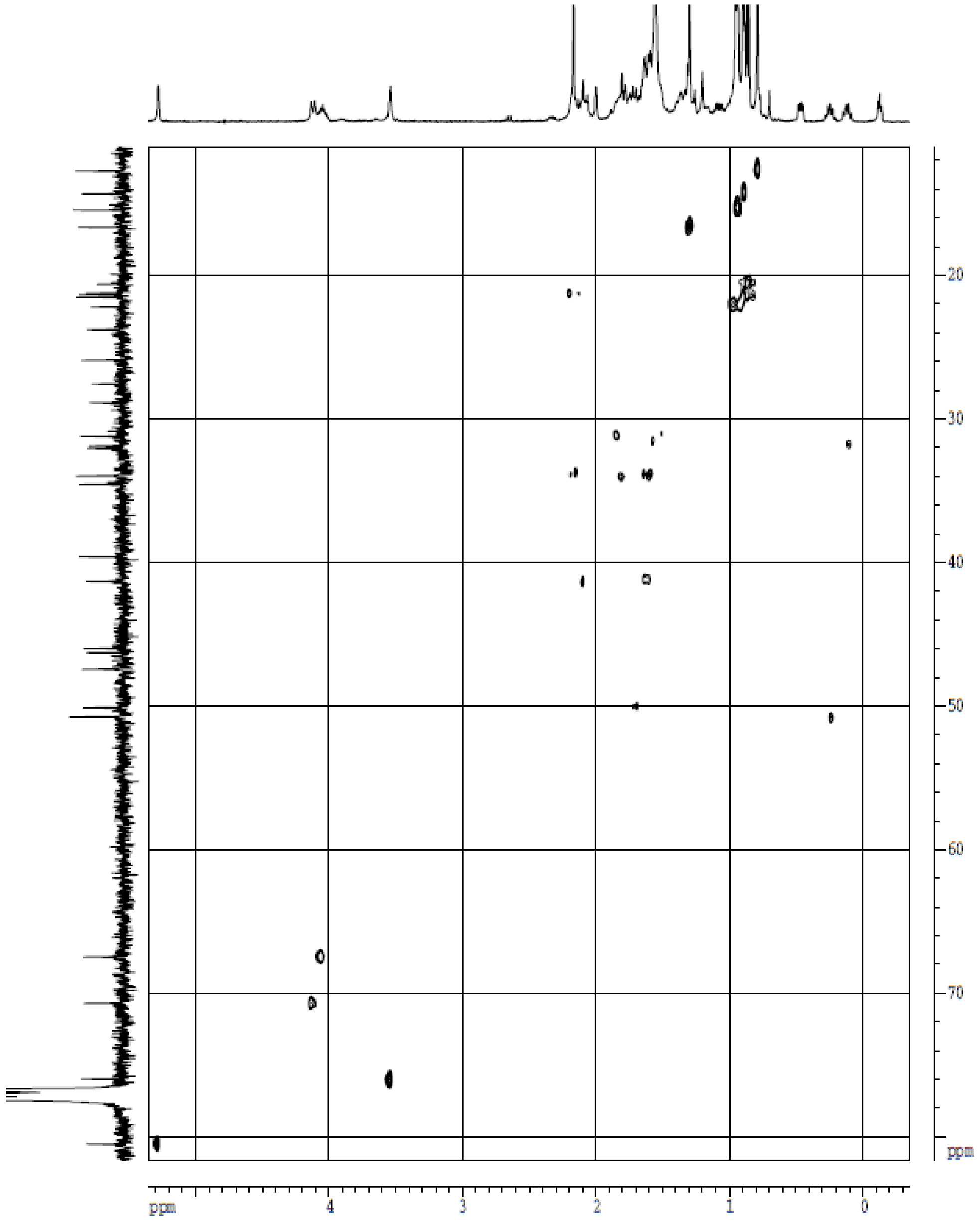

Figure S55. ^{1}H-^{1}H COSY spectrum of the new compound **7**.

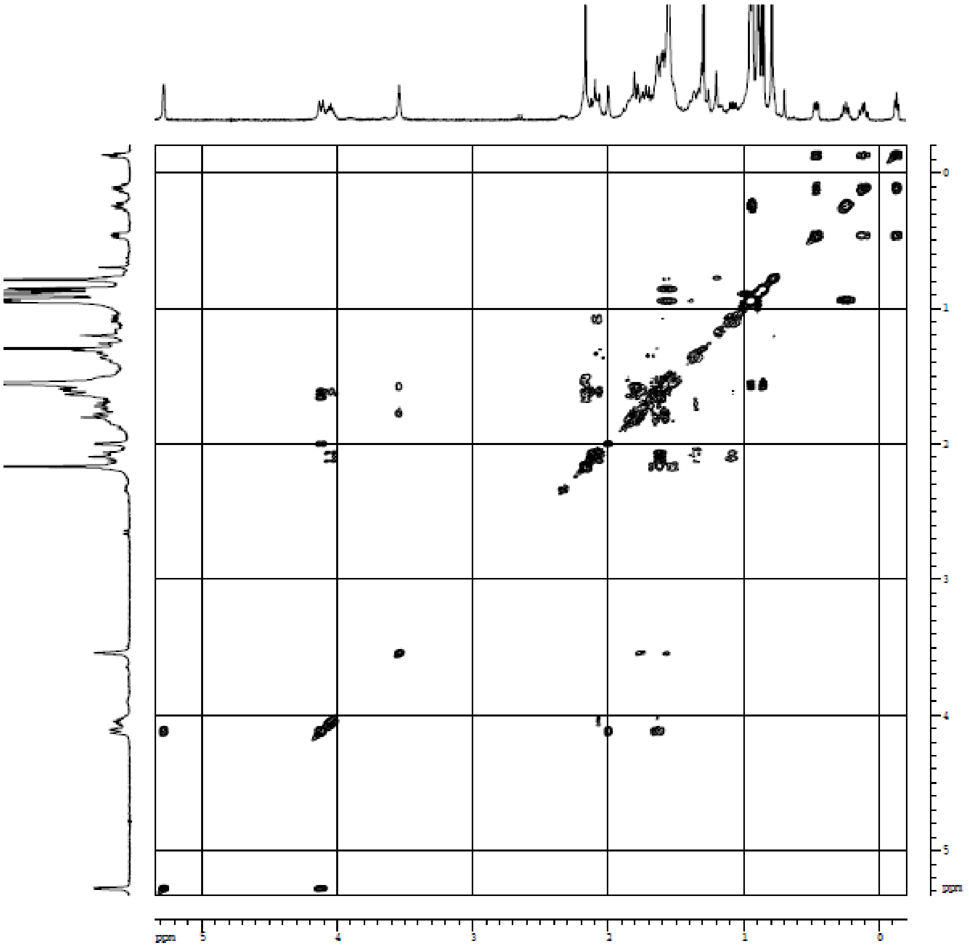

Figure S56. HMBC spectrum of the new compound **7**.

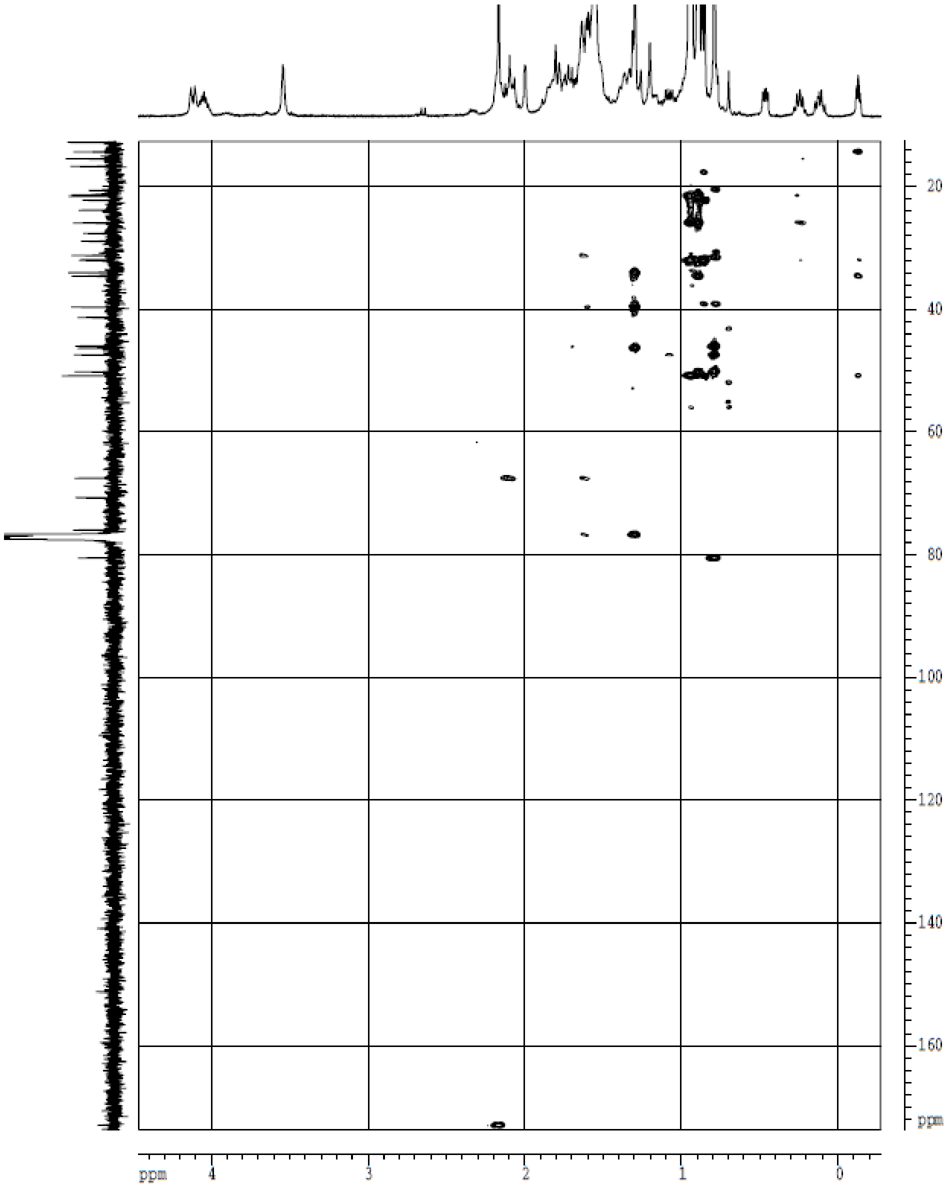

Figure S57. NOESY spectrum of the new compound **7**.

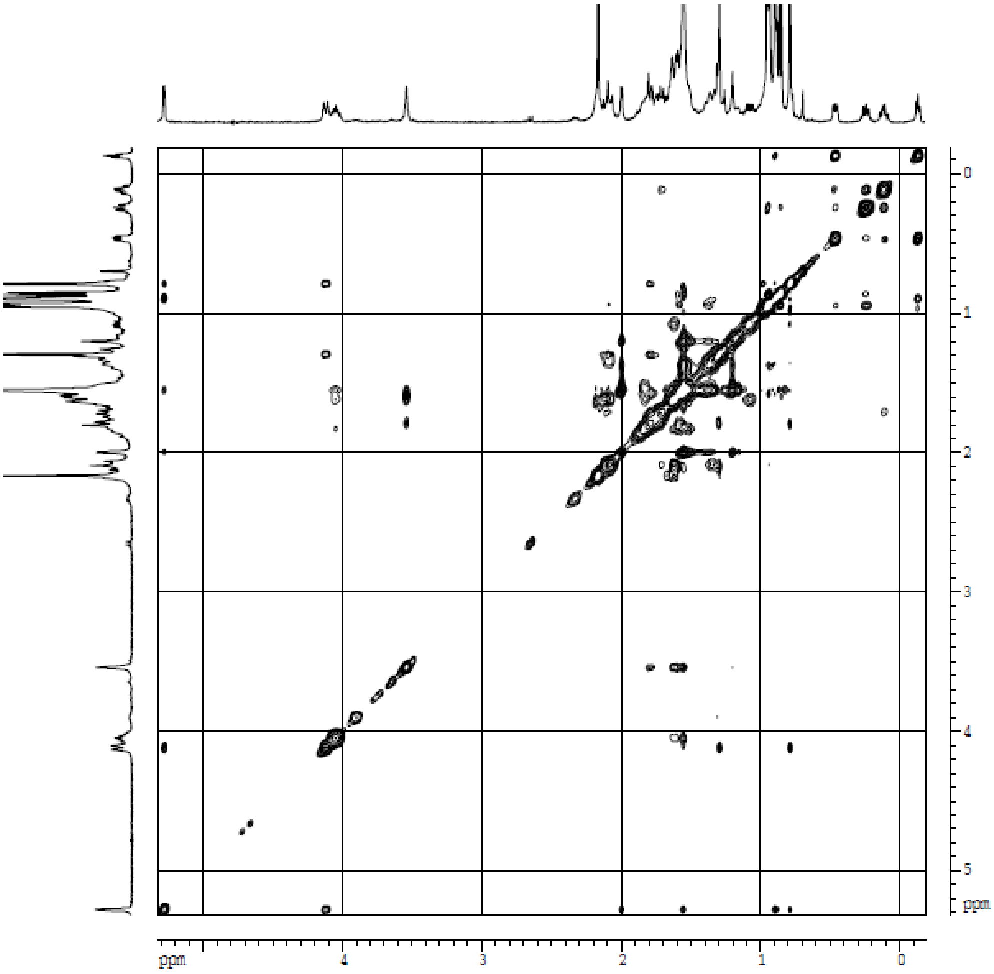

Supplement: Supplementary File 1 — Supplementary Materials (PDF, 2362 KB) [file marinedrugs-11-00775-s001.pdf]
